# Neuropeptides as transmitters and regulators of the locus coeruleus noradrenergic system

**DOI:** 10.1016/j.pharmr.2025.100081

**Published:** 2025-07-02

**Authors:** Léa J. Becker, Madison M. Martin, Alex C. Hughes, Bernard Mulvey, Chao-Cheng Kuo, Sean C. Piantadosi, Michael R. Bruchas, Keri Martinowich, Frank J. Meye, Lindsay A. Schwarz, David Weinshenker, Jordan G. McCall, Danai Riga

**Affiliations:** 1 Department of Anesthesiology, Washington University in St. Louis, St. Louis, Missouri; 2 Center for Clinical Pharmacology, Washington University School of Medicine, St. Louis, Missouri; 3 Washington University Pain Center, Washington University in St. Louis, St. Louis, Missouri; 4 Department of Anesthesiology and Pain Medicine and Department of Pharmacology, Center for the Neurobiology of Addiction, Pain and Emotion, University of Washington, Seattle, Washington; 5 Department of Medical Oncology, University of Colorado Anschutz, Aurora, Colorado; 6 Lieber Institute for Brain Development, Johns Hopkins Medical Campus, Baltimore, Maryland; 7 Department of Psychiatry and Behavioral Sciences, Johns Hopkins School of Medicine, Baltimore, Maryland; 8 The Solomon H. Snyder Department of Neuroscience, Johns Hopkins School of Medicine, Baltimore, Maryland; 9 Johns Hopkins Kavli Neuroscience Discovery Institute, Baltimore, Maryland; 10 Department of Translational Neuroscience, Brain Center, UMC Utrecht, Utrecht University, Utrecht, The Netherlands; 11 Department of Developmental Neurobiology, St. Jude Children’s Research Hospital, Memphis, Tennessee; 12 Department of Human Genetics, Emory University School of Medicine, Atlanta, Georgia; 13 Department of Human Genetics, Amsterdam University Medical Center (VUmc), Amsterdam, The Netherlands; 14 Department of Functional Genomics, Center for Neurogenomics and Cognitive Research (CNCR), Vrije Universiteit Amsterdam, Amsterdam, The Netherlands

## Abstract

The locus coeruleus (LC), the brain’s main source of noradrenaline, has received increased attention due to the recently unveiled heterogeneity of its cell types. Departing from the long-standing idea of molecular, anatomical, and functional uniformity of the structure, we now understand the LC as a multiplexed nucleus, capable of temporally precise and targeted neuromodulation of distinct brain regions and functions. The LC neuropeptidergic landscape provides a window into this remarkable neuronal diversity. Stemming from recent technological advances that have allowed for LC transcriptional profiling, a wealth of data on the (co)expression of LC neuropeptides and their cognate receptors has come to light. Peptidergic systems are ideally situated to exert neuromodulatory control over the LC noradrenergic system. This peptidergic control can occur both locally, within the LC and the neighboring peri-LC microcircuitry, and at distal LC terminal fields. The functional significance of LC neuropeptidergic signaling in physiological processes and pathological conditions is an emerging field. Here we compile existing literature on the expression, anatomical distribution, physiological effects, and, when available, behavioral role of the major neuropeptidergic populations of, and innervating, the LC and peri-LC. Furthermore, we highlight current methodologies that delineate LC peptidergic input/output, aiming at uncovering their functional role. Finally, we discuss how neuropeptidergic signaling enables LC modularity and thus sustains a multifaceted role of physiological noradrenaline release dynamics with a rich feature set of behavioral representations.

**Significance Statement:**

The locus coeruleus (LC) noradrenergic system influences a variety of neurophysiological processes to coordinate complex behaviors. These far-reaching neuromodulatory effects are not solely mediated by noradrenaline, but rather, by a variety of coreleased neuropeptides that can alter postsynaptic responses in LC terminal regions. In addition, the LC itself is regulated by a multitude of incoming peptidergic signals that drive wide-ranging changes in LC neuronal physiology and the subsequent patterns of noradrenaline release. It is important to understand how neuropeptide (co)transmitters and regulators of the LC can drive circuit-level plasticity and adaptive behavioral responses to changing environmental stimuli. This review compiles our current understanding of these processes, providing additionally crucial insights into the mechanisms underlying LC dysfunction and its many related neuropsychiatric conditions.

## I Introduction

The locus coeruleus (LC) is a small noradrenergic nucleus located in the brainstem, adjacent to the fourth ventricle. It extensively innervates the central neuraxis, serving as the primary source of noradrenaline (NA)-mediated neuromodulation.^1^ Owing to its brain-wide ascending and descending collaterals, which allow for both global/diffused release and discrete/targeted NA-mediated signaling,^2^^,^^3^ the LC coordinates a host of basic physiological and high-order neurocognitive processes. These range from regulation of autonomic responses such as respiration and heart rate during sleep/arousal cycles,^4^ to sensory processing,^5^ approach-avoidance conflicts,^6^^,^^7^ learning, memory,^8^^,^^9^ and decision-making under cognitively demanding conditions.^10^ Accordingly, allostatic changes in LC function substantially alter information relay throughout the brain, and can lead to a diverse array of behavioral maladaptations and associated psychopathology, including stress-related, attentional and impulse control disorders.^11^, ^12^, ^13^

Behavioral arousal levels are mediated by tonic and phasic firing patterns of LC neurons. The LC has a baseline tonic firing rate of 1–2 Hz,^14^, ^15^, ^16^, ^17^ which is elevated to ∼5–6 Hz during hyperarousal states such as stress and anxiety and suppressed during hypoarousal states such as sleep and anesthesia.^14^^,^^18^^,^^19^ LC neurons also exhibit phasic activity of up to 15 Hz in response to salient or task-relevant stimuli,^15^^,^^18^^,^^20^^,^^21^ which is thought to contribute to the output of task-related behaviors. Recently, the diversity in LC-NA neuronal firing modes has been expanded further than these 2 states to suggest these firing patterns occur across a continuous manifold shaped by different species, sexes, and recording modalities.^22^ Historically, LC activity has thought to exist with an inverted U-shaped relationship between arousal and task performance. This curve follows the classical Yerkes-Dodson model, which states that optimal performance occurs at moderate arousal levels, whereas performance is impaired when arousal is too high or too low.^23^ This relationship is driven by LC firing patterns, with moderate tonic activity and prominent phasic activity contributing to optimal arousal levels and task performance.^10^^,^^24^ Thus, the LC can act as a regulator of many behavioral outputs through gain control of downstream cortical and subcortical pathways.

Largely defined by NA expression,^25^ the LC has been traditionally viewed as a homogeneous structure that indiscriminately broadcasts global gain control signals related to general wakefulness or attentiveness during salient events (eg, upon novelty or stress).^26^ With the advancement of cell type–specific and projection-specific manipulations, and the development of intersectional molecular techniques, it is now apparent that the LC system includes substantial anatomical and functional diversity. In recent years, several comprehensive reviews have further delineated this diversity,^2^^,^^3^^,^^27^ leading to the conceptualization of a heterogeneous LC-NA system capable of temporally precise, targeted physiological responses to ever-changing environments. In this review, we aim to build upon this emerging diversity by emphasizing another source of LC multiplicity that relies on its complex neurochemical makeup. Particularly, single-cell and -nucleus sequencing datasets^28^^,^^29^ have revealed that, besides putative NA neurons, the LC and its immediate surroundings (ie, the peri-LC) are composed of a wide assortment of NA-coexpressing or -lacking cell clusters distinctly defined by their peptidergic content. Together with the discovery of a multitude of neuropeptidergic receptors, located either on NA neurons or neighboring cells, this has created a fertile ground for discerning how LC neuronal activity is modulated and how this peptidergic signaling influences output in ways not yet considered.

Neuromodulatory peptidergic signaling, mediated primarily by G-coupled protein receptors (GPCRs), initiates downstream intracellular cascades that markedly impact cell membrane properties, neuronal excitability, and synaptic strength.^30^ Factoring in volume transmission, the impact of peptide-mediated neurophysiological changes are expansive in both spatial and temporal dimensions, reaching receptors up to 1 mm from the original release site, and operating on fast-acting as well as long-lasting time scales.^31^ Together, peptidergic neuromodulation of the LC and its efferent targets can trigger persistent circuit-level plasticity, ultimately impacting physiology and related behavioral representations.

The sheer number of peptide/receptor combinations present in this region argues in favor of a multimodal network that fine-tunes LC activity on demand. To facilitate understanding of the functional outcomes of GPCR-mediated neuromodulation of the LC-NA system, we summarize studies whereby LC neuropeptides and their cognate receptors have been investigated. We discuss local and distal peptidergic inputs that exert neuromodulatory influence over the LC and describe the GPCRs that mediate their effects on NA cell physiology and resulting patterns of NA release. Furthermore, we detail the role of peptides that are coreleased by LC fibers at afferent sites, and how they act synergistically to (or independent from) NA to drive discrete behaviors. In addition, we highlight new technological developments that have enabled a nuanced study of neuropeptidergic signaling in the LC, viewing advantages and pitfalls of each methodology. Finally, we put forward remaining questions that we consider fundamental for the advancement of understanding the functional features of this critical brain-wide neuromodulatory system.

## II Locus coeruleus peptides and cognate receptors

**Fig. 1:**
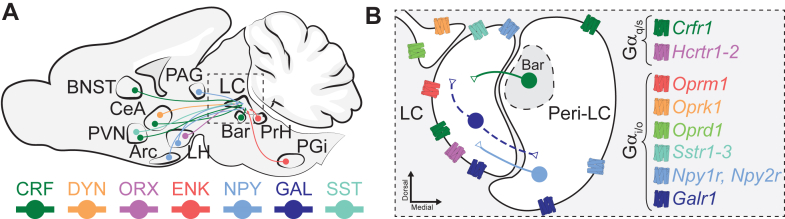
Primary neuropeptidergic cotransmitters and regulators of the LC. (A) Schematic representation of the primary neuropeptidergic afferents to the LC. CRF (green) afferents originate from the BNST, CeA, PVN, and Barrington’s nucleus (Bar). DYN (orange) afferents originate from the CeA. Orexinergic (ORX; purple) inputs originate from the lateral hypothalamus (LH). Enkephalinergic (ENK; red) afferents originate from the nucleus paragigantocellularis (PGi) and nucleus prepositus hypoglossi (PrH). NPY (light blue) afferents originate from the PVN, the arcuate nucleus (Arc), the LH and the periaqueductal gray (PAG). Galaninergic (GAL; dark blue) inputs originate from the LC itself. SST (cyan) afferents originate from the PVN. Dotted outline square indicates the LC proper and peri-LC region. (B) Zoomed-in schematic of LC and the peri-LC (including Barrington’s nucleus) highlighting additional local peptidergic inputs and G_*α*q/s_ and G_*α*i/o_ coupled receptors. CRF is released from Barrington’s nucleus, NPY is released by peri-LC neurons, and GAL is believed to be co-released from LC-NA neurons locally affecting LC and peri-LC neurons (blue dotted line). Receptors are color coded according to their highest affinity ligand based on inputs from A and referred to by their gene names. Corticotropin-releasing factor receptor 1 (green; *Crfr1*), Orexin A receptor 1 and Orexin B receptor (purple; *Hcrtr1 and Hcrtr2*, respectively), *μ*-opioid receptor (red; *Oprm1*), *κ*-opioid receptor (orange; *Oprk1*), δ-opioid receptor (light green; *Oprd1*), somatostatin receptors 1-3 (cyan; *Sstr1-3*), neuropeptide Y receptor 1 and 2 (light blue; *Npy1r* and *Npy2r*), and galanin receptor 1 (dark blue; *Galr1*).

The LC adheres to a tight spatial organization,^32^ divided into the LC proper, a nuclear core that is exclusively composed of noradrenergic cell bodies,^33^ and the peri-LC dendritic zone, a site of input integration from LC afferents.^34^ The LC proper represents the primary source of LC neurochemical diversity, with large or sparse peptide coexpressing subpopulations distributed among noradrenergic cells, that are capable of peptidergic corelease in afferent-specific manners. Likewise, the dendritic pericerulean space, typically including the Barrington’s, (medial) parabrachial, and laterodorsal tegmental nuclei, is an area of rich neurochemical composition that receives peptidergic inputs from widely distributed efferent regions. A plethora of peptide-containing neuronal subpopulations reside in the peri-LC, among an enriched cluster of GABAergic interneurons^35^ that regulate LC-NA-mediated arousal responses.^36^ Below we review key studies on the LC neuropeptide cotransmitters and regulators from studies that focus on these 2 LC compartments (Fig. 1).

### A Insights from recent RNA sequencing data

**Fig. 2:**
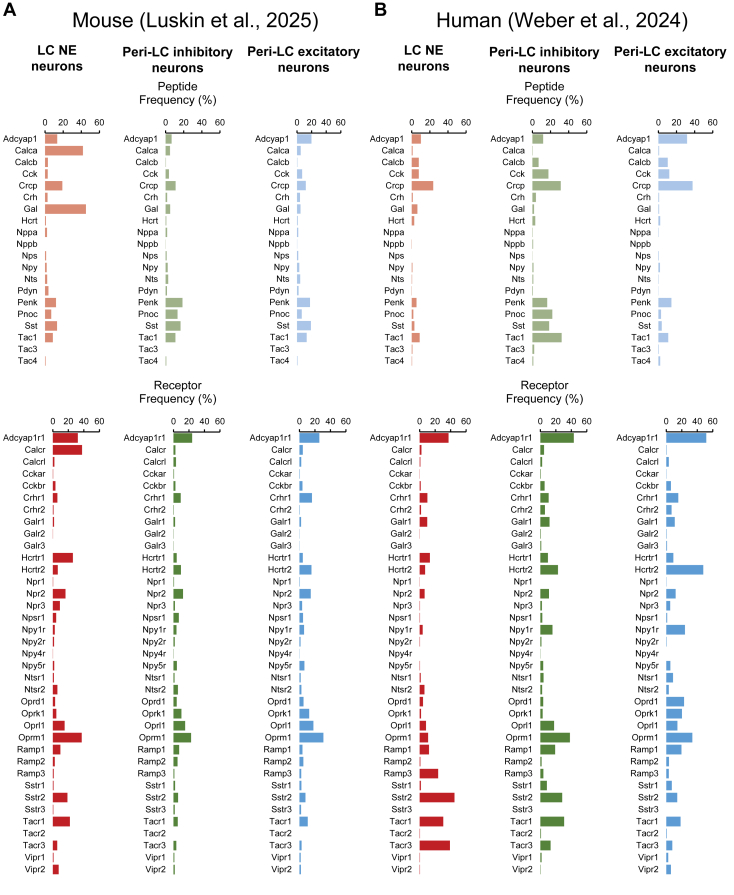
Transcriptomic profiling of neuropeptide and receptor expression in the LC and peri-LC of mice and humans. A graphical summary of RNAseq data from Luskin et al^29^ (mouse) and Weber et al^37^ (human) depicting the percentage of (A) noradrenergic LC neurons, (B) inhibitory peri-LC neurons, and (C) excitatory peri-LC neurons expressing select neuropeptides and their cognate receptors. *Calca*, calcitonin related polypeptide alpha; *Calcr*, calcitonin receptor; *Crf*, corticotropin-releasing factor; *Gal*, galanin; *Hcrtr*, hypocretin receptor; *Nps*, neuropeptide S; *Npy*, neuropeptide Y; *Oprk*, opioid receptor kappa; *Oprl*, opioid related nociceptin receptor; *Oprm*, opioid receptor mu; *Pdyn*, prodynorphin; *Penk*; proenkephalin; *Pnoc*, prepronociceptin; *Sst*, somatostatin; *Tac*, tachykinin.

The development of RNA sequencing (RNAseq) technologies for single-cell transcriptomic profiling alongside new spatial in situ and RNAseq methods has provided unique opportunities to thoroughly characterize the molecular composition and anatomical arrangement of gene expression in any brain region. We describe below how RNAseq has been used in recent years to identify an abundance of neuropeptide and related receptor genes enriched within the mouse and human LC (Fig. 2).^37^

#### 1 Mice

Several transcriptomic studies have used RiboTag^38^ or translating ribosome affinity purification (TRAP) techniques to bulk sequence RNAs bound to LC-specifically labeled ribosomes. The first focused examination of the mouse LC transcriptome reported >3000 genes to be highly expressed in adulthood, including genes encoding neuropeptides and their receptors. In particular, the genes for Galanin (*Gal*), calcitonin receptor (*Calcr*), orexin/hypocretin receptor type 1 (*Hcrtr1*), and somatostatin receptor 2 (*Sstr2*) were shown to be enriched in the LC compared with the hindbrain.^39^ This study also evaluated differentially expressed genes across sex and found that tachykinin receptor 3 (*Tacr3*) gene expression was more highly upregulated in the LC of female mice compared to males. Interestingly, no significant sex differences were observed in corticotropin-releasing factor receptor 1 (*Crfr1*) or *μ* opioid receptor 1 (*Oprm1*) gene expression, although prior studies have identified sex differences in behavioral and physiological responses to CRF-1 and *μ*-opioid receptor (*μ*-OR) agonists.^40^, ^41^, ^42^, ^43^ More recently, TRAP has been leveraged to investigate transcriptome-wide responses of the mouse LC to injury by N-(2-chloroethyl)-N-ethyl-2-bromobenzylamine (DSP-4),^44^ a neurotoxin selective for noradrenergic axons originating from the LC, and in response to human tyrosinase transgene-driven production of neuromelanin.^45^

Two recent studies have used single-cell/-nucleus RNAseq (snRNAseq) to more thoroughly characterize expression of neuropeptides and their receptors in the mouse LC at ages 21–28 days, which corresponds to early adolescence,^28^ and in the LC and peri-LC of adult mice.^29^ Both groups identified abundant *Gal* and calcitonin (*Calca*) gene expression in noradrenergic LC neurons, along with smaller proportions of proenkephalin (*Penk*), prodynorphin (*Pdyn*), somatostatin (*Sst*), and tachykinin precursor 1 (*Tac1*). These 2 reports were inconclusive regarding the extent of *Pdyn* expression, and only Luskin et al^29^ detected expression of the genes for prepronociceptin (*Pnoc*) and neuropeptide S (*Nps*). In terms of peptide receptor expression, both studies reported expression of *Calcr*, *Oprm1*, nociceptin receptor 1 (*Oprl1*), orexin/hypocretin receptors type 1 and 2 (*Hcrtr1* and *Hcrtr2*), tachykinin receptors 1 and 3 (*Tacr1* and *Tacr3*), somatostatin receptors 2 and 3 (*Sstr2* and *Sstr3*), corticotropin-releasing factor receptors 1 and 2 (*Crfr1* and *Crfr2*), galanin receptor 1 (*Galr1*), neuropeptide Y receptors 1 (*Npy1r*), 2 (*Npy2r*) and 5 (*Npy5r*), along with, to a variable extent, neuropeptide S receptor 1 (*Nps1r*).

Neuropeptide expression in peri-LC GABAergic and glutamatergic neurons was further addressed in one study^29^ (Fig. 2). A large proportion of GABAergic peri-LC cells expressed *Penk*, *Sst*, *Pnoc*, and *Tac1*, along with lower levels of *Calca*, tachykinin precursor 2 (*Tac2*), *Npy*, *Crf*, *Hcrt*, and *Nps*. Glutamatergic peri-LC neurons also showed relatively high levels of *Penk* and *Sst* expression, along with moderate levels of *Tac1* and *Pnoc* and low levels of *Crf*, *Pdyn*, and *Nps*. Excitatory and inhibitory peri-LC populations each expressed several neuropeptide receptors, including *Oprm1*, *Oprl1*, *κ* opioid receptor 1 (*Oprk1*), *Crfr1*, *Hcrtr1*, *Hcrtr2*, *Sstr2*, *Npsr1*, *Tacr1*, *Tacr3*, *Npy1r*, *Npy2r*, and *Npy5r*. These results indicate that the LC proper and peri-LC dendritic zone are composed of genetically heterogeneous neuropeptidergic subpopulations, which may contribute to the functional diversity of the LC-NA system.

Both single-cell studies above also described the spatial distributions of neuropeptide transcripts within the LC region using fluorescent in situ hybridization (FISH), and one additionally used polony-indexed library-sequencing^46^ to identify spatially resolved RNA transcripts. As expected, genes for the catecholaminergic biosynthetic enzyme tyrosine hydroxylase (*Th*), dopamine *β*-hydroxylase (*Dbh*), and *Gal* were shown to be densely expressed in the LC proper with both methods. Polony-indexed library-sequencing further demonstrated broadly distributed *Calca*, *Hctr1*, *Nps*, *Npy*, *Penk*, *Pnoc*, *Sst*, and *Tac1* expression across the LC and peri-LC. Greater *Nps*, *Penk*, and *Sst* expression was reported in the posterior peri-LC, including a distinct *Sst* cluster ventromedial to the LC.^29^ FISH characterizations of *Slc32a1* (the gene encoding for the vesicular GABA transporter; VGAT)-expressing peri-LC cells revealed coexpression of *Penk*, *Pnoc*, and *Tac1*,^29^ along with sparse *Nps*, *Npy*, and *Galr1* expression^28^ consistent with the RNAseq data. RNAseq and in situ methods were inconclusive with regard to *Npy1r*, *Npy2r*, and *Npy5r* expression in *Th*^+^ populations, likely due to intrinsic challenges of collecting a large number of cells from a small structure like the mouse LC, which is composed of only ∼2000 neurons.^47^ The sheer quantity and variety of neuropeptides and receptors within the LC region suggest a host of possible discrete receptor interactions which specify LC-NA activity rates under changing conditions, but the limitations of interpreting molecular data with functional consequences remain. We address this topic more in detail later in this review.

#### 2 Humans

The small size and positioning of the LC deep within the human brainstem has rendered it relatively intractable to comprehensive molecular characterization; thus, transcriptomic profiling studies in this region remain limited. The Allen Brain Atlas first acquired data that included the LC using laser microdissection and RNA microarray assays in a study that assayed hundreds of finely defined brain areas.^48^ A second multitissue initiative, FANTOM5, collected LC samples from 3 adult brain donors and 1 infant brain donor and performed Cap Analysis of Gene Expression sequencing, which quantifies transcripts arising from promoters and enhancers.^49^ Notably, such readouts approximate gene expression and noncoding distal regulator (eg, enhancer) activities.^50^ FANTOM5 remains the only available functional genomic study of human LC. Later, the Human Protein Atlas produced bulk RNAseq from postmortem human brain tissue punches, including LC from 7 donors.^51^

More recently published studies using snRNAseq and spatially resolved transcriptomics represent a major advance in our understanding of the molecular landscape of the human LC and surrounding anatomical regions.^37^ Weber et al^37^ performed spatially resolved transcriptomics on tissue determined to contain LC by FISH (*Th* and the noradrenergic transporter *Slc6a2*) from 4 neurotypical adult human brain donors, along with snRNAseq from adjacent tissue from 3 of the same donors. snRNAseq collected using fluorescence-assisted nucleus sorting to enrich neuronal nuclei from tissue cryosections yielded transcriptomes from putative LC-NA neurons and local GABAergic and glutamatergic populations. The LC and the immediately surrounding inhibitory and excitatory neuron clusters of the peri-LC expressed various neuropeptides and receptors; overall, the proportions of each population expressing a given transmitter or receptor was relatively consistent between mice and humans, with the notable exception that human LC (unlike mouse) was practically devoid of both *Calca* and *Calcr* (Fig. 2).

Spatial transcriptomic data from Weber et al^37^ highlights the promise of these evolving molecular methods for improving the yield of LC, which has been the critical obstacle to its molecular characterization. Visium (10x Genomics) was used to collect tissue on slides with thousands of ∼50 *μ*m diameter RNA capture spots, which have unique x,y coordinate sequence tags incorporated during sequencing preparation. These spatial tags enable mapping detected genes onto H&E histology images collected from the same tissue. By pairing microscopy images to the gene expression spots, the LC was annotated based solely on its distinctively large cells/nuclei and neuromelanin pigmentation prior to gene expression quantification. LC spots annotated visually were enriched (relative to the rest of the tissue) for 32 genes including expected LC-NA markers, ie, *Dbh*, *Slc6a2*, *Th*, and *Slc18a2*. Measuring gene expression from intact, imaged tissue holds promise for improving the efficiency and accuracy of collecting LC from human tissue. With the continued rapid evolution of spatial transcriptomic technologies, well powered studies of the human LC/peri-LC structural organization in relation to gene expression and neuropeptidergic diversity will become feasible. It will be particularly important and interesting to examine these expression profiles in human disease states and preclinical models thereof. Despite limited sample size, the Weber data provide an important initial resource for interrogating the human LC and peri-LC at the level of individual cells and open doors to new explorations into cellular heterogeneity, cross-species conservation, and peptide neurotransmission in this important brain area. As these types of datasets grow and we begin to compare naïve brains to pathological states, new therapeutic targets may emerge for the treatment of LC-related neurological and neuropsychiatric diseases.

#### 3 Resources for transcriptome-wide results from human and mouse locus coeruleus

The Allen Brain Atlas human microarray data continues to be provided as part of its sizable collection of interactive tools. These data can be searched interactively at https://human.brain-map.org/ and the original microarray results can be obtained at http://human.brain-map.org/static/download. FANTOM5 reprocesses its Cap Analysis of Gene Expression sequencing results to keep alignments and transcript counts consistent with current reference genomes; both current and previous iterations of these results (RNAseq read alignment files in BAM format and peak coordinates in BED format) are available for download at https://fantom.gsc.riken.jp/5/datafiles/reprocessed/hg38_latest. Links to additional resources including University of California, Santa Cruz browser sessions for their data can be found at https://fantom.gsc.riken.jp/5/. Human Protein Atlas bulk RNAseq can be queried through their portal (https://www.proteinatlas.org/), with results per donor and region for a particular gene; additionally, processed gene and transcript expression profiles can be downloaded at https://www.proteinatlas.org/about/download#brain. Interactive web tools for exploring gene expression in the Weber et al^37^ study of neurotypical adult human LC are available for their single-nucleus (https://libd.shinyapps.io/locus-c_snRNA-seq/) and Visium spatial transcriptomic results, and both processed datasets are available in the R package *WeberDivechaLCdata* (https://bioconductor.org/packages/release/data/experiment/html/WeberDivechaLCdata.html).

Several transcriptome-wide profiles of mouse LC are also available to the public. A RiboTag study in 2014 generated microarray profiles of LC and other cell types in part of a larger study comparing gene expression between (otherwise) wild-type and *Mecp2* knockout (KO) animals at 2 ages;^38^ corresponding triplicates of microarray data from wild-type adult and young LC are in Gene Expression Omnibus (GEO) (full accession GSE8720; adult wild-type accessions GSM215871-215873; young wild-type accessions GSM1464137-1464139). In addition, these microarray data are incorporated into Neuroexpresso (http://www.neuroexpresso.org), an interactive web tool for visualization of microarray results from dozens of cell type–specific gene expression studies in the mouse brain.^52^ The LC expression data from Mulvey et al^39^ are available through GEO (accession GSE100005), and the study’s supplemental files provide transcriptome-wide results from analyses of cell type–specific and sex-differential gene expression. TRAP data from mouse LC expressing neuromelanin or after treatment with DSP-4 can be found at GSE226827 and GSE221404, respectively. Single-cell RNAseq datasets from adult mouse LC and peri-LC described by Luskin et al*^29^* are available under GEO accession GSE201998, and single-nucleus results adolescent mouse LC in Caramia et al^28^ are available under GEO accession GSE236349.

### B Types of peptidergic transmission in the (peri-) locus coeruleus

**Fig. 3:**
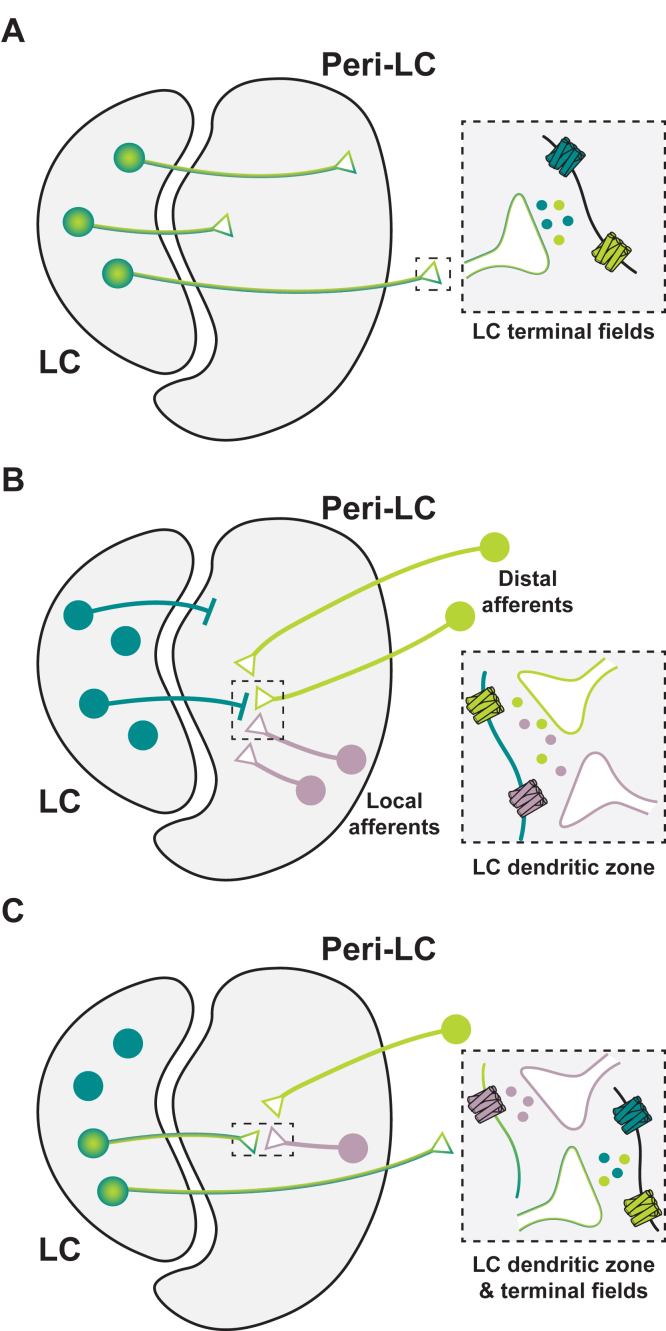
Types of neuropeptidergic signaling in the LC and peri-LC space. Based on existing literature, 3 types of NA-neuropeptide interactions are expected in the LC and peri-LC. (A) Type 1: LC-NA neurons capable of coreleasing neuropeptides influence postsynaptic cells expressing adrenergic and/or peptidergic receptors at LC terminal fields. (B) Type 2: LC noradrenergic cells that express neuropeptidergic receptors are modulated by peptidergic input arriving from local or distal sources. (C) Type 3: LC-NA cells that express both a neuropeptide and its receptor relay dual signals to postsynaptic targets *and* are modulated by incoming peptidergic input. *Cyan*: NA neurons/axon terminals/adrenergic receptors/NA; *Green* (distal) *or purple* (local): neuropeptidergic neurons/peptidergic receptors/peptide. *Cyan-Green*: NA/neuropeptide coexpressing neurons/axon terminals.

Considering the functional implications of neuropeptide systems in the LC and peri-LC, there are likely 3 major types of peptidergic transmission involving the LC-NA system, demarcated by peptide expression and receptor localization in the area: **type 1)** when a neuropeptide but not its receptor is expressed by LC noradrenergic neurons, influencing postsynaptic target cells by NA/peptide cotransmission; **type 2)** when a neuropeptide receptor but not the neuropeptide itself is expressed by LC noradrenergic neurons, enabling LC modulation by local (ie, originating within the peri-LC) or distal peptide-containing afferents; and **type 3)** when a neuropeptide *and* its receptor are expressed by LC noradrenergic neurons, where the LC can both influence through and be influenced by the neuropeptide. This last scenario could also indicate the existence of a local feedback mechanism for the control and regulation of LC activity (Fig. 3).

There are numerous examples of positive and negative feedback mechanisms that govern neuronal activity, represented by the canonical relationship between NA and the *α*2-adrenergic receptor (reviewed in the study by Huang et al^53^). Upon stimulation, LC neurons release NA somatodendritically, acting through autocrine/paracrine transmission on local *α*2-autoreceptors to suppress LC firing and further NA release.^54^ Although peptidergic transmission types 1 and 2 have been shown conclusively for many neuropeptides and their receptors, type 3 has not and currently exists only as an important testable hypothesis. In Galanin, we review the evidence for and against, and the challenges of investigating, the existence of such a mechanism (Table 1). The physiological and behavioral functions of several widely studied LC-related neuropeptides follow, presented by alphabetical order.

**Table 1:** Literature summary of the neuropeptidergic modulation of LC-NA neurons activity

Ligand	Dose, Route	Species	Sex	LC Activity	Recording Technique	Detailed Results	PMID
***CRF***							
CRF	5–41 ng, puff	Rat	Males	↑	Whole cell recording, acute slice	↑firing, blocked by CP154,526 (CRH1R antagonist)	15509759
CRF	10–200 ng, bath	Rat	Males	Variable	Whole cell recording, acute slice	↑ (50 ng) or ↓ (200 ng) sEPSC amplitude, half-with and charge transfer, no effect (10, 25, 100 nM)	27973694
CRF	300 nM, bath	Mouse	Both	↑	Whole cell recording, acute slice	↑firing, blocked by CBr1-KO	30103253
CRF	100 mM, bath	Mouse	Both	No effect/↓ glutamatergic inputs	Whole cell recording, acute slice	↓oEPSC (PAG), no effect (PFC, LH)	36156249
CRF	10 *μ*M	Mouse	both	↑	Imaging recording, acute slice	↑ FQ of calcium spikes	38895281
CRF	0.3–3 mg i.c.v.	Rat	Males	↑	In vivo single unit recordings	↑firing, enhanced by cold exposure	11239906
CRF	0.1–100 ng, intra-LC infusion	Rat	Both, intact and gonadectomized	↑	In vivo single unit recordings	↑firing (females > males), potentiated by acute stress	16123744
CRF	3 *μ*g i.c.v.	Rat	Males	↑	In vivo single unit recordings	↑firing, reduced by acute or chronic stress	7777167
CRF	0.03–3 *μ*g i.c.v.	Rat	Males	↑	In vivo single unit recordings	↑firing, stress-induced sensitization (low doses) or desensitization (higher doses)	10336508
CRF	3–100 ng, intra-LC infusion or i.c.v.	Rat	males	↑	In vivo single unit recording	↑firing (intra-LC>i.c.v.), blocked by DPheCRF_12–41_	9103494
CRF	300 ng, i.c.v.	Rat	Males	↑	In vivo single unit recording	↑firing, blocked by Astressin and NBI-35965	16095571
CRF	3 ng, intra-LC infusion	Rat	Males	↑	In vivo single unit recording	↑firing, potentiated by morphine exposure	15385601
CRF	0.3–3 *μ*g, i.c.v.	Rat	Males	↑/no effect	In vivo single unit recording	↑firing (1–3 *μ*g), no effect (0.3 *μ*g)	3492683
Ala14 CRF (inactive analog)	3 *μ*g, i.c.v.	Rat	Males	No effect	In vivo single unit recording	No effect	3492683
CRF	0.5 nM, i.c.v. or intra-LC infusion	Rat	Males	↑/↓	In vivo single unit recording	↑firing, ↓sensory-evoked firing	6603889
CRF-OH	0.5 nM, i.c.v.	Rat	Males	No effect	In vivo single unit recording	No effect	6603889
CRF	3 *μ*g, i.c.v.	Rat	Males	↑	In vivo single unit recording	↑firing	8301577
DPheCRF_12-41_	0.03–3 *μ*g i.c.v.	Rat	Males	No effect	In vivo single unit recording	No effect alone, block CRF-induced ↑firing	8301577
*α*hCRF	10–50 *μ*g i.c.v.	Rat	Males	No effect	In vivo single unit recording	Block CRF-induced ↑firing	8301577
CRF	3 *μ*g/*μ*L i.c.v.	Rat	Males	↑	In vivo multiple unit recordings	↑firing, stress-induced desensitization	8813346
CRF	0.5 *μ*g, intra-LC infusion	Rat	Both	↑	In vivo multiple unit recordings	↑firing LC and PVN	16930446
CRF	1/3 *μ*g i.c.v.	Rat	Males	↑	In vivo single and multiple unit recordings	↑firing, strain dependent (1 *μ*g)	9452188
CRF	3–100 ng, intra-LC infusion	Rat	Males	↑	Microdialysis	↑NA release in PFC	9103494
CRF	3 *μ*g, i.c.v.	Rat	Males	↑	Microdialysis	↑NA release in anterior hypothalamus	8431775
CRF	0.6–14.4 *μ*g intra-HPC infusion	Rat	Males	↑/↓	Microdialysis	↑ (1.44–7.2 *μ*g) or ↓ (14.4 *μ*g) NA release in HPC	8058208
CRF	0.17–0.51 nM, intra-LC infusion	Rat	Males	↑	Microdialysis	↑NA and MHPG in parietal cortex, blocked by *α*hCRF	8738583
CRF	100 ng, intra-LC infusion	Rat	Males	↑	Microdialysis	↑NA and MHPG in PFC, blocked by *α*hCRF	7882052
CRF	2 *μ*g, i.c.v.	Rat	Males	↑	Microdialysis	↑NA release in PFC	7982086
*α*hCRF	50 *μ*g, i.c.v.	Rat	Males	No effect	Microdialysis	No effect alone, block stress-induced ↑NA release in PFC	7982086
CRF	2/20/60 ng, intra-LC infusion	Rat	Males	↑	Microdialysis and in vivo single unit recording	↑firing (LC), ↑NA release in HPC	10421711
DPheCRF_12-41_	3 *μ*g, intra-LC infusion	Rat	Males	No effect	Microdialysis and in vivo single unit recording	No effect alone, block stress-induced ↑ LC firing and ↑NA release in HPC	10421711
CRF	1/10 *μ*g i.c.v.	Rat	Males	↑/no effect	HPLC-ECD	↑ MOPEG/NA ratio in HPC and PFC, no effect on NAc, STR and AMY	2785246
CRF	0.5–10 *μ*g i.c.v.	Rat	Males	↑	Fluorometric	↑MHPG-SO_4_ in hypothalamus, AMY, LC, pons and medulla	8327547
CRF	20–100 ng, intra-LC infusion	Rat	Males	↑	In vivo amperometry	↑NA release in HPC	12231226
CRF	100 ng, intra-LC infusion	Rat	Males	↑/↓	In vivo voltametry	Small transient ↓NA release in HPC followed by robust ↑NA release in HPC	10704782
CP-154,526	1–5.6 mg/kg, i.v.	Rat	Males	No effect	In vivo single unit recording	No effect alone, block CRF-induced ↑firing	8816826
CP-154,526	100 *μ*M, intra-LC infusion	Rat	Males	No effect	Microdialysis	No effect alone, block stress-induced ↑NE release in PFC	10650173
DPheCRF_12-41_	3 *μ*g, i.c.v.	Rat	Males	No effect	In vivo single unit recording	No effect alone, block stress-induced ↑firing	22210331
DPheCRF_12-41_	3 *μ*g, i.c.v. or 10 ng, intra-LC infusion	Rat	Males	No effect	In vivo single unit recording	No effect alone, block CRD-induced ↑firing	9187321
*α*hCRF	1.2 *μ*g, intra-LC infusion	Rat	Females	No effect	In vivo single unit recording	No effect alone, block IL-1-induced LC inhibition	12372984
*α*hCRF	150 ng, intra-LC infusion	Rat	Males	No effect	In vivo single unit recording	No effect alone, block nitroprusside-induced ↑firing	8152620
*α*hCRF	150 ng, LC infusion or 50 *μ*g, i.c.v.	Rat	Males	No effect	In vivo single unit recording	No effect alone or on sensory-evoked LC activity, block nitroprusside-induced ↑firing	1933327
*α*hCRF	1 *μ*g, intra-LC infusion	Rat	males	No effect	Microdialysis	No effect alone, block stress-induced ↑NE release in PFC	9128917
CeA^-LC^ CRF^+^ fibers photostimulation	N/A	Mouse	Males	No effect	Whole cell recording	No effect on EPSC/IPSC	26212712
CeA^-LC^ CRF^+^ fibers photostimulation	N/A	Mouse	Males	↑	16-channel array in vivo	↑firing	26212712
***Galanin***							
Galanin	1 nM–1 *μ*M, bath; 10 pM puff	Rat	Males	↓	Whole cell recording, acute slice	↓firing, ↓Vm, ↓Rm, ↑K^+^ conductance, outward current, desensitization	7538638
Galanin	250 nM, bath	Rat	Males	↓	Whole cell recording, acute slice	↓firing, ↓Vm, ↑GIRK channel conductance, PTX-sensitive G_i/o_ protein activation	29852172
Galanin	1–100 nM, bath	Rat	Males	↓	Extracellular recording, acute slice	↓firing (14/19 neurons)	2474450
Galanin	1–10 nM, bath	Rat	Males	↓	Whole cell and extracellular recording, acute slice	↓firing, ↓Vm	7678551
Galanin	1–1 *μ*M, bath	Rat	Males	↓	Whole cell recording, acute slice	↓firing, ↓Vm (38/38 neurons)	11689176
AR-M961, (GalR1/R2 agonist)	1 nM–1 *μ*M, bath	Rat	Males	↓	Whole cell recording, acute slice	↓firing, ↓Vm (42/42 neurons), ↑K^+^ conductance	11689176
AR-M1896 (GalR2 agonist)	100 nM–1 *μ*M, bath	Rat	Males	↓	Whole cell recording, acute slice	↓firing, ↓Vm (26/34 neurons)	11689176
M617 (GalR1 agonist)	500 nM, bath	Mouse	Both	↓	Whole cell recording, acute slice	↓firing, ↑Kv	37487094
***Melanin/melanin concentrating hormone (MCH)***							
MCH	100 ng, intra-LC infusion	Rat	Males	↓	HPLC-ECD	↓NA in PFC	38135278
***NPY***							
NPY	0.1 *μ*M, bath	Rat	N/A	↓	Whole cell recording, acute slice	↑NA-induced IPSC	1436133
NPY	0.1 *μ*M, bath	Rat	N/A	↓	Whole cell recording, acute slice	↓firing, ↓Rm, ↑NA hyperpolarization	1968229
NPY	0.1 *μ*M, bath	Rat	N/A	↓	Whole cell recording, acute slice	↑NA hyperpolarization	8114954
NPY (16-36)	0.1 *μ*M, bath	Rat	N/A	↓	Whole cell recording, acute slice	↑NA hyperpolarization	8114954
PYY	0.1 *μ*M, bath	Rat	N/A	↓	Whole cell recording, acute slice	↑NA hyperpolarization	8114954
[Leu31, Pro34]NPY	0.1 *μ*M, bath	Rat	N/A	No effect	Whole cell recording, acute slice	No effect	8114954
NPY	30 nM, bath	Mouse	Males	↓	Whole cell recording, acute slice	↓firing, blocked by BIBO-3304	40700482
NPY	300 nM, bath	Mouse	Males	↑	Whole cell recording, acute slice	↑firing, blocked by BIIE-0246 or ionotropic receptor antagonists	40700482
NPY	Endogenous (chemogenetically evoked release)	Mouse	Both	↓	Whole cell recording, acute slice	↓firing, ↓Vm, blocked by BIBO-3304	40700482
NPY	2 nM, i.c.v.	Rat	Males	No effect	In vivo microdialysis	No effect alone, ↑KCl-induced NA release in PVN	9098556
[Leu31, Pro34]NPY	2 nM, i.c.v.	Rat	Males	↓	In vivo microdialysis	↓NA release in PVN, ↑KCl-induced NA release in PVN	9098556
NPY, GR231118 (NPY Y1 receptor antagonist)	0.05–0.1 *μ*M, in vitro superfusion	Rat	Males	↓	HPLC-ECD	↓NA in the hypothalamus (0.10 *μ*M), blocked by GR231118	11164766
NPY, [Leu31,Pro34]NPY	0.1 *μ*M, in vitro superfusion	Rat	Males	↓	HPLC	↓NA in the hypothalamus and medulla, ↓DHPG in the medulla	15177927
***Opioids***							
***Delta***							
DPDPE (δ-OR agonist)	1 *μ*M, bath	Rat	Both	↑	Whole cell recording, acute slice	↑ firing, ↓ m- & eIPSCs in LC^→SC^ neurons	12424303
SNC-162 (δ-OR agonist)	10 *μ*M, bath	Mouse	Both	No effect	Imaging recording, acute slice	No effect on FQ of calcium spikes	38895281
Overexpression of δ-OR in LC	N/A	Rat	Males	↓	Whole cell recording, acute slice	↓ stress-induced increase of LC firing rate	35884666
***Kappa***							
DYN	200 nM, bath	Mouse	Both	↓glutamatergic inputs/no effect	Whole cell recording, acute slice	↓ oEPSC from LH and PAG. No effect on PFC inputs	36156249
U50488 (*κ*-OR agonist)	100 nM, bath	Rat	Males	N/A	Whole cell recording, acute slice	↓eEPSC	3031541
U50488 (*κ*-OR agonist)	10, 30, 100 ng, intra-LC infusion	Rat	Both	↓	In vivo multiple unit recording	↓sensory stimuli-evoked phasic activty	18562623
CI-977 (*κ*-OR agonist)	1 nM, bath	Rat	Both	N/A	Whole cell recording, acute slice	↓eEPSC	1315940
U-50488 (*κ*-OR agonist)	10 *μ*M, bath	Mouse	Both	No effect	Imaging recording, acute slice	No effect on FQ of calcium spikes	38895281
norBNI (*κ*-OR antagonist)	2.5 *μ*g/side, intra-LC infusion	Mouse	Males	↓	IHC	↓pERK	23787819
***Mu***							
ENK	0.01–100 *μ*M, bath	Rat P10-P21	Both	N/A	Whole cell recording, acute slice	↓Ca^2+^conductance	10455306
Met-ENK	0.3, 30 *μ*M, bath	Rat	Both	N/A	Whole cell recording, acute slice	Outward current, ↓effect with morphine pretreatment, ↓desensitization in phosphorylation-deficient MOR cells	32362586
Met-ENK	1, 10 *μ*M, bath	Rat	Both	N/A	Whole cell recording, acute slice	Outward current, enhanced by forskolin	8890275
Met-ENK	20 mM, intra-LC iontophoresis	Rat	Both	N/A	Whole cell recording, acute slice	Outward current	7472359
Met-ENK	0.3, 30 *μ*M, bath	Rat	Males	N/A	Whole cell recording, acute slice	Outward current, ↑desensitization with muscarine	8778299
Met-ENK	30 *μ*M, bath	Rat	Males	N/A	Whole cell recording, acute slice	Outward current, ↑desensitization by PKC activator	15361548
Met-ENK	10, 30 *μ*M, bath	Rat	Males	N/A	Whole cell recording, acute slice	Outward current	1915781
Met-ENK	30 *μ*M, bath	Mouse	Both	N/A	Whole cell recording, acute slice	Outward current (wild-type), no effect (GIRK2/GIRK3^-/-^)	18702733
Met-ENK	0.3, 3, 30 *μ*M, bath	Mouse	Both	N/A	Whole cell recording, acute slice	Outward current	18612077
Met-ENK	30 *μ*M, bath	Mouse	Both	N/A	Whole cell recording, acute slice	Outward current (wild-type), no effect (GIRK2/GIRK3^–/–^)	12040038
Met-ENK	30 *μ*M, bath	Mouse	Both	N/A	Whole cell recording, acute slice	Outward current, ↓desensitization in phosphorylation-deficient MOR cells	30664663
Met-ENK	30 *μ*M, bath	Mouse	Both	N/A	Whole cell recording, acute slice	Outward current, ↑recovery of NA-induced outward current after met-ENK application (β-arr-2 KO)	22689562
Met-ENK	0.8, 3 *μ*M, bath	Rat	Males	↓	Whole cell and extracellular recording, acute slice	↓firing;], outward current, ↑desensitization by nitric oxide	22593094
Met-ENK	0.3, 30 *μ*M, bath	Rat	Males	N/A	Whole cell recording and fluorescence imaging	Outward current; ↓internalization by kinase inhibitor, ↑receptor recovery by p38MAPK inhibitor or GRK infusion	22113080
Met-ENK	0.3, 30 *μ*M, bath	Rat	Males	↓	Whole cell recording, acute slice	↓firing, ↓Vm	1651377
Met-ENK	0.03–30 *μ*M, bath	Rat	Both	N/A	Whole cell recording, acute slice	Outward current, less effective in fentanyl-treated but not oxycodone-treated animal	35193934
Met-ENK	0.1–30 *μ*M, bath	Rat	Males	N/A	Whole cell recording, acute slice	Outward current	7582522
Met-ENK	30 *μ*M, bath	Rat	Males	N/A	Whole cell recording, acute slice	Outward current, ↑desensitization (PKC activator), ↓desensitization (PKC inhibitor)	19200236
Met-ENK	0.3, 30 *μ*M, bath	Rat	Males	N/A	Whole cell recording, acute slice	Outward current (control), no effect (morphine-treated)	22914548
Met-ENK	30 *μ*M, bath	Rat P10-21	Both	N/A	Whole cell recording, acute slice	Outward current, ↓eIPSC	11917119
Met-ENK	10 *μ*M, bath	Mouse	Both	N/A	Whole cell recording, fluorescence imaging, cell culture	↓Vm, ↑internalization	16611829
Met-ENK	30 *μ*M, bath	Rat	Males	N/A	Whole cell recording, acute slice	Outward current, ↓desensitization (ERK inhibitor)	26013542
Met-ENK	0.8, 3 *μ*M, bath	Rat	Males	↓	Extracellular recording, acute slice	↓firing	26254861
Met-ENK	0.8 *μ*M, bath	Rat	Males	↓	Extracellular recording, acute slice	↓firing	38035000
Met-ENK	0.03, 0.1 *μ*M, bath	Rat	Males	↓	Extracellular recording, acute slice	↓firing	7678551
Leu-ENK	10 *μ*M, bath	Rat P22-29	Both	↓	Whole cell recording, acute slice	↓firing, ↓Vm, outward current	22284180
Endomorphin 1	3 *μ*M, bath	Rat P10-21	Both	N/A	Whole cell recording, acute slice	Outward current	11917119
Endomorphin 2	10 *μ*M, bath	Rat	Males	N/A	Whole cell recording, acute slice	Outward current, ↓desensitization (ERK inhibitor)	26013542
Dermorphin	0.1, 1 *μ*M, bath	Mouse	Both	N/A	Whole cell recording, fluorescence imaging, cell culture	↓Vm, ↑internalization	16611829
Normorphine (agonist)	0.001–3 *μ*M, bath	Rat	Males	N/A	Whole cell recording, acute slice	Outward current	7582522
DAMGO (*μ*-OR agonist)	0.1, 3 *μ*M, bath	Rat	Males	↓	Whole cell recording, acute slice	↓firing, ↓Vm	1651377
DAMGO (*μ*-OR agonist)	0.01–100 *μ*M, bath	Rat	Males	N/A	Whole cell recording, acute slice	Outward current	7582522
DAMGO (*μ*-OR agonist)	10 *μ*M, bath	Rat	Males	N/A	Whole cell recording, acute slice	Outward current, ↑desensitization (PKC activator), ↓desensitization (PKC inhibitor)	19200236
DAMGO (*μ*-OR agonist)	0.1–10 *μ*M, bath	Rat P10-21	Both	N/A	Whole cell recording, acute slice	Outward current	11917119
DAMGO (*μ*-OR agonist)	10 *μ*M, bath	Rat	Males	N/A	Whole cell recording, acute slice	Outward current, ↓desensitization (ERK inhibitor)	26013542
DAMGO (*μ*-OR agonist)	1, 10 *μ*M, bath	Mouse	Males	↓	Whole cell recording, acute slice	↓firing	31430399
DAMGO (*μ*-OR agonist)	1 *μ*M, bath	Mouse	Both	↓	Extracellular recording, acute slice	↓firing	37961541
DAMGO (*μ*-OR agonist)	1 *μ*M, bath	Rat	Both	↓	Field potential recording, acute slice	↓rhythmic field potential	29447951
DAMGO (*μ*-OR agonist)	200 nM, bath	Mouse	Both	↓	Imaging recording, acute slice	↓ FQ of calcium spikes	38895281
DAMGO (*μ*-OR agonist)	0.1–30 pg, intra-LC infusion	Rat	Both	↓	In vivo single unit recording	↓firing, more effective in males	27827371
DAMGO (*μ*-OR agonist)	0.5 nM–5 *μ*M, bath	Guinea pig, rat	Males	↓	Radioactivity of [3H]NA release, acute slice	↓NA release	28211438
Morphine (agonist)	1 *μ*M, bath	Rat	Males	N/A	Whole cell recording, acute slice	Outward current (control), no effect (morphine-treated)	22914548
Morphine (agonist)	30 *μ*M, bath	Rat	Males	N/A	Whole cell recording, acute slice	Outward current, ↓desensitization (ERK inhibitor)	26013542
Morphine (agonist)	1 *μ*M, bath	Rat	Males	↓	Whole cell recording, acute slice	↓firing, ↓input resistance	3967768
Morphine (agonist)	1 *μ*M, bath	Rat	Both	N/A	Whole cell recording, acute slice	Outward current, less effective in fentanyl-treated but not oxycodone-treated animal	35193934
Morphine (agonist)	30 *μ*M, bath	Rat	Males	N/A	Whole cell recording, acute slice	Outward current, ↑desensitization (PKC activator), ↓desensitization (PKC inhibitor)	19200236
Morphine (agonist)	30 *μ*M; 0.1–9.6 mg/kg, i.c.v.	Rat	Males	↓	Whole cell recording, acute slice & *in vivo* single unit recording	↓firing, outward current, ↑desensitization in morphine-treated or CCI model	30284123
Morphine (agonist)	50 *μ*g/h, i.c.v., 1 and 7 days	Rat	Males	↓	*in vivo* single unit recording	↓firing, potentiate CRF effect	15385601
Morphine (agonist)	0.5–32 mg/kg, i.p	Mouse	Both	↓	In vivo single unit recording	↓firing, less effective in GIRK2 KO	23040084
Morphine (agonist)	1 *μ*g intra-LC infusion	Cat	Both	↓	In vivo single unit recording	↓firing	3380325
CYLE (photoactivatable MOR agonist)	10 *μ*M, bath, with 5 ms, 1–91 mW UV illumination	Rat P22-29	Both	↓	Whole cell recording, acute slice	↓firing, ↓Vm, outward current	22284180
Methadone (agonist)	1 *μ*M, bath	Rat P10-21	Both	N/A	Whole cell recording, acute slice	Outward current	11917119
Naloxone (antagonist)	1 *μ*M, bath	Rat	Males	N/A	Whole cell recording, acute slice	Inward current (morphine-treated)	22914548
Naloxone (antagonist)	5 *μ*M,bath	Rat	Males	No effect/ ↑	Whole cell recording, acute slice	No effect (control), ↑firing and ↑Vm (morphine-treated)	11387385
Naltrexone (antagonist)	100 *μ*M, bath	Rat	Males	No effect	Whole cell recording, acute slice	No effect (control), ↑firing (morphine-treated)	1618268
Naloxone (antagonist)	1 *μ*g intra-LC infusion	Cat	Both	No effect	In vivo single unit recording	No effect on firing	3380325
Naloxone (antagonist)	2 mg/kg, i.p.	Rat	Both	No effect/ ↑	In vivo single unit recording	No effect (control), ↑firing (morphine-treated)	33892002
Naltrexone (antagonist)	1 *μ*g i.c.v.	Rat	Males	No effect/ ↑	In vivo single unit recording	No effect, ↓phasic activity evoked by sensory stimuli	2743108
CNV-NLX (photoactivatable MOR antagonist)	5 *μ*M, bath, with 0.1–5 s, 10 mW, 405 nm illumination	Rat	Both	↓	Whole cell recording, acute slice	Attenuate outward currents driven by MOR agonists	23960100
***Nociceptin***							
N/OFQ	3–300 nM, bath	Rat	Males	N/A	Whole cell recording, acute slice	↓Ca^2+^conductance, outward current	10588934
N/OFQ	3–300 nM, bath	Rat	Males	N/A	Whole cell recording, acute slice	Outward current	1915781
N/OFQ	2.5 nM, bath	Mouse	Both	↓	Imaging recording, acute slice	↓FQ of calcium spikes	38895281
N/OFQ	50 nM, bath	Mouse	Both	↓	Extracellular recording, acute slice	↓firing	37961541
NCNH_2_ (NOPR agonist)	10 pM–10 *μ*M, bath and intra-LC infusion	Rat	Males	↓	In vivo and in vitro microdialysis	↓NA release in cerebral cortex & cortical slice	11323113
Nphe1 (NOPR antagonist)	100 *μ*M, bath and intra-LC infusion	Rat	Males	N/A	In vivo and in vitro microdialysis	Block NCNH_2_ effect	
***Orexin/hypocretin***							
ORXA	100 nM, bath	Rat	Males	N/A	Whole cell recording, acute slice	Enhances the percentage of decrease in MetEnkephalin-induced GIRK current	28385341
ORXA	100 nM, 1 *μ*M, bath	Rat	Males	No effect/↑	Amperometry, acute slice	No effect (100nM) ↑NA release (1 *μ*M)	18354023
ORXA	Intra-LC infusion, iontophoresis	Rat	Males	↑	In vivo single unit recording	↑firing	11027239
ORXA	100 *μ*M, intra-LC infusion	Rat	Males	↑	In vivo single unit recording	↑firing during rest period	18614159
ORXA	1, 10, 100 nM	Rat	Males	↑	In vivo microdialysis	↑NA release in dentate gyrus	15329388
ORXA	0.04–5 nM i.c.v.	Rat	Males	↑	In vivo microdialysis	↑NA release in PFC	19007758
ORXA	50 nM, bath	Mouse	Both	↑	Imaging recording, acute slice	↑ FQ of calcium spikes	38895281
SB-334867-A (ORXA antagonist)	100 *μ*M, intra-LC infusion	Rat	Males	↓	In vivo single unit recording	↓firing during active period no effect during rest period	18614159
ORXB	0.1–100 *μ*M, bath	Rat	Males	↑	Whole cell recording, acute slice	↑firing, ↑Vm	10545156
ORXB	1 *μ*M bath, 50–100 mM puff	Rat	Males	↑	Whole cell recording. acute slice	↑firing, ↑Vm	10852238
ORXA/ORXB	1 *μ*M, bath	Mouse E18-19	Both	↑	Whole cell recording, cell culture	↑firing, ↑Vm	12015428
ORXA/ORXB	1 *μ*M, bath	Mouse P0-5	Both	↑	Whole cell recording, acute slice	↑firing, ↑Vm	
ORXA/ORXB	0.3–3 *μ*M (ORXA), 1–10 *μ*M (ORXB), bath	Rat/mouse	Males	↑	Extracellular recording, acute slice	↑firing (ORXA>>ORXB)	
ORXA/ORXB	100 *μ*M i.c.v.	Rat	Males	↑	In vivo single unit recording	↑firing	11353017
PeFLH stimulation	N/A	Rat	Both	↑	In vivo single unit recording	↑firing, blocked by SB-334867	24311996
ORX neuron ablation	N/A	Mouse	Males	N/A	Whole cell recording. acute slice	↓sIPSC frequency	23922890
				↑	In vivo single unit recording	↑firing	
***SST***							
SST	30 nM, bath	Rat	Both	↓	Whole cell recording, cell culture	↓firing, ↓Vm	12702704
SST	2 nM–10 *μ*M, bath	Rat	Both	↓	Whole cell recording, acute slice	Outward current	9422800
SST	10 *μ*M, bath	Rat	Both	N/A	Whole cell recording, acute slice	Outward current	35193934
SST	10 *μ*M, bath	Rat	Both	N/A	Whole cell recording, acute slice	Outward current	32362586
SST	0.01–1 *μ*M, bath	Rat	Both	N/A	Whole cell recording, acute slice	Outward current	2476550
SST	1–100 nM, bath	Gerbil	Males	↓	Whole cell and extracellular recording, acute slice	↓firing, ↓Vm	2448659
SST	0.1 nM–10 *μ*M, bath	Rat	Males	↓	Extracellular recording, acute slice	↓firing	8732275
SST	20 nM,bath	Mouse	Both	↓	Imaging recording, acute slice	↓ FQ of calcium spikes	38895281
Cortistatin (agonist)	2 nM–10 *μ*M, bath	Rat	Both	↓	Whole cell recording, acute slice	Outward current	9422800
Sandostatin (agonist)	5–100 nM, bath	Rat	Males	↓	Whole cell recording, acute slice	↓firing, ↓Vm	8190003
***Substance P (SP)***							
SP	3 *μ*M, puff	Mouse P1-5	Both	Variable	Whole cell recording, cell culture	↑firing, ↑Vm (5/17 neurons), ↓firing, ↓Vm (4/17 neurons), no effect (8/17 neurons)	2430074
SP	0.03–3 *μ*M, bath	Rat	N/A	↑	Whole cell recording, acute slice	↑Vm, ↓Kir conductance, ↑cations conductance, inward current	1279460
SP	0.3 *μ*M, puff	Rat	Both	Variable	Whole cell recording, cell culture	Outward current, ↓Kir conductance; inward current, ↑nonselective ionic conductance; mixed response.	7506620
SP	0.01–1 *μ*M, bath	Rat	Males	↑	Extracellular recording, acute slice	↑firing	6197310
SP	1–100 nM, bath	Gerbil	Males	↑	Extracellular recording, acute slice	↑firing	2448659
SP	100 nM, bath	Guinea pig	Males	↑	Extracellular recording, acute slice	↑firing	11823070
SP	200 nM, bath	Mouse	Both	↑	Imaging recording, acute slice	↑ FQ of calcium spikes	38895281
SP	2.75 mM, intra-LC iontophoresis	Rat	Males	↑	In vivo single unit recording	↑firing	412562
SP O-methyl ester	0.3 *μ*M	Guinea pig	Males	↑	Whole cell recording, acute slice	↑firing, blocked by NK1R antagonist CP 96,345 (*μ*M)	7509748
[SAR9,Met(O2)11]-SP (NK1R agonist)	10 mM, intra-LC infusion	Guinea pig	Males	↑	In vivo microdialysis	↑NA release in PFC, blocked by NK1R (GR 205171) and NK3R antagonist (SR142801)	11746734
Septide (NK1R agonist)	1 mM, intra-LC infusion	Guinea pig	Males	↑	In vivo microdialysis	↑NA release in PFC	11746734
L-760735 (NK1R antagonist)	100 nM, bath	Guinea pig	Males	No effect	Extracellular recording, acute slice	No effect	11823070
L-760735 (NK1R antagonist)	Chronic, 3 mg/kg daily for 4 weeks, oral	Guinea pig	Males	↑	Whole cell and extracellular recording, acute slice	↑ burst firing	11823070
CP-96,345 (NK1R antagonist)	0.15 mg/kg, i.v.	Rat	Males	No effect	In vivo single unit recording	No effect alone, attenuate clonidine-induced LC inhibition	10817615
WIN-51,708 (NK1R antagonist)	2 mg/kg, i.v.	Rat	Males	No effect	In vivo single unit recording	No effect alone, attenuate clonidine-induced LC inhibition	10817615
CP-96,345 (NK1R antagonist)	10 mg/kg, 2 days treatment i.p.	Rat	Males	No effect	In vivo single unit recording	No effect alone, attenuate clonidine-induced LC inhibition	18930727
CP-99,994 (NK1R antagonist)	10 mg/kg, 2 days treatment i.p.	Rat	Males	No effect	In vivo single unit recording	No effect alone, attenuate clonidine-induced LC inhibition	18930727
CP-99,994 (NK1R antagonist)	10 mg/kg, 14 days treatment s.c.	Rat	Males	No effect	In vivo single unit recording	No effect alone, attenuate clonidine-induced LC inhibition	18930727

AMY, amygdala; *β*-arr-2, *β*-arrestin-2; CBr1, cannabinoid receptor 1; CCI, chronic constriction injury; CRD, colorectal distention; DHPG, 3,4-dihydroxyphenylglycol; DPDPE, D-Pen2,D-Pen5 enkephain; E, embryonic day; e/m/o/sEPSC/IPSC, evoked/miniature/spontaneous excitatory postsynaptic currents/inhibitory postsynaptic currents; HPC, hippocampus; HPLC-ECD, high-performance liquid chromatography with electrochemical detection; i.c.v., intracerebroventricular; IHC, immunohistochemistry; IL-1, interleukin-1; Kir, inwardly rectifying potassium; MHPG, 3-methoxy-4-hydroxyphenylglycol; MHPG(-SO4), 3-methoxy-4-hydroxyphenylethylenegiycol (sulfate); MOPEG, 3-methoxy-4-hydroxy-phenylglycol; N/OFQ, nociceptin/orphanin FQ; NAc, nucleus accumbens; NK(1/3)R, neurokinin receptor 1/3; norBNI, norbinaltorphimine; P, postnatal day; PAG, periaqueductal grey; PeFLH, perifornical area in the posterior lateral hypothalamus; PFC, prefrontal cortex; PTX, pertussis toxin; PVN, paraventricular nucleus; PYY, peptide YY; Rm, membrane resistance; SC, spinal cord; STR, striatum; Vm, membrane potential.

### C Corticotropin-releasing factor

Corticotropin-releasing factor (CRF, also known as corticotropin-releasing hormone) was first isolated in the early 1980s and has been well established as a regulator of the hypothalamic-pituitary-adrenal (HPA) axis in response to stress.^55^^,^^56^ However, CRF’s actions have also been demonstrated outside the HPA axis. This extrahypothalamic CRF is known to activate neural circuits involved in stress-induced anxiety, aversion, and reinstatement of drug-seeking behavior, independent of HPA activation.^57^, ^58^, ^59^, ^60^ Although numerous questions remain regarding how CRF interacts with the noradrenergic system, many studies have reported exciting developments regarding the inputs providing CRF to the LC, the effects of endogenous and exogenous CRF on LC neuron physiology, and the role of LC CRF in mediating anxiety- and stress-related behaviors.

#### 1 Anatomy

The LC receives sparse CRF projections, primarily from local peri-LC sources such as the Barrington’s nucleus, as well as the nucleus paragigantocellularis.^61^, ^62^, ^63^ Conversely, the peri-LC receives dense CRF projections from the paraventricular nucleus of the hypothalamus (PVN), the central amygdala (CeA), and the bed nucleus of the stria terminalis (BNST).^61^^,^^63^, ^64^, ^65^, ^66^ The aforementioned studies specifically focused on the lateral peri-LC, finding CRF axons to be mostly localized to the rostrolateral region. CRF terminal expression in the medial peri-LC has not been as extensively characterized, although it has been determined that it, together with the LC core, receives CRF projections from the CeA.^6^ Within the rostrolateral peri-LC, CRF^+^ terminals from the PVN, CeA, and BNST form synaptic contacts with tyrosine hydroxylase (TH)–labeled dendrites.^64^, ^65^, ^66^ These contacts likely indicate that the LC receives CRF from the PVN, CeA, and BNST via projections anatomically situated in the peri-LC and the broad dendritic field of the LC itself. Notably, BNST terminals form more symmetric (inhibitory) synapses with TH-labeled dendrites, whereas PVN and CeA terminals form more asymmetric (excitatory) synapses.^64^, ^65^, ^66^ Combined with evidence suggesting that a smaller proportion of BNST terminals in the rostrolateral peri-LC are CRF^+^ compared to PVN or CeA terminals,^64^, ^65^, ^66^ it is possible that CRF from the BNST has distinct effects on the regulation of LC activity through selective interactions with noradrenergic LC neuronal ensembles. Whether this drives subsequent behavior has yet to be tested directly using high resolution circuit-based approaches.

CRF^+^ terminals in the LC also overlap with other peptidergic projections. Electron microscopy data support broad colocalization of CRF and neuropeptide Y (NPY) axon terminals (∼32%) within the peri-LC dendritic zone,^67^ albeit the origins of dual-peptidergic LC projectors remain unidentified. Likewise, LC CRF^+^ terminals coincide with the endogenous opioids enkephalin (ENK) and dynorphin (DYN). A small subset of ENK^+^ terminals that form synaptic contacts with TH-labeled dendrites coexpress CRF, whereas a relatively larger subset of DYN+ terminals in the LC coexpress CRF.^68^^,^^69^ A substantial portion of DYN^+^ neurons in the CeA coexpress CRF, and likewise over half of CRF^+^ neurons in the CeA coexpress DYN,^69^ suggesting that the CeA may be a prominent source of CRF^+^/DYN^+^ terminals in the LC. However, the source of the overlap between CRF and ENK is less clear. It has been noted that 30% of PVN terminals are CRF^+^, whereas only 2% are ENK^+^,^64^ and that lesioning the CeA reduces labeling of CRF but not ENK in the LC,^68^ together suggesting that most of the overlap between CRF and ENK expression comes from another source. Intersectional strategies that, for example, employ viral tracing in cell clusters defined by their peptidergic content will clarify the origins of such dual-peptidergic input to the LC and the peri-LC area.

#### 2 Receptors and physiology

CRF exerts its effects by binding to 2 distinct G_S_ protein-coupled receptors: CRFR-1 and CRFR-2. In situ hybridization studies have had inconsistent results regarding the presence of CRFR mRNA in the LC. Several rodent studies failed to detect either CRFR-1 or CRFR-2 mRNA in LC neurons,^70^, ^71^, ^72^ whereas one study in the rat LC detected CRFR-1 mRNA expression that was elevated in response to forced walking stress.^73^ However, multiple reports using other methods including RNAseq, immunostaining, radiolabeling, and reverse transcription polymerase chain reaction (RT-PCR) have identified CRFR-1 expression in LC neurons of both rodents and nonhuman primates,^28^^,^^29^^,^^74^, ^75^, ^76^ though some suggest that LC CRFR-1 expression is low and largely restricted to the peri-LC dendritic field.^77^^,^^78^ CRFR-2 expression was detected in the LC by RNAseq^28^^,^^29^ but not radiolabeling,^75^ illustrating differential resolution in the 2 methodological approaches.

Extensive evidence has demonstrated that CRF has an excitatory effect on the LC-NA system (Table 1). In vitro direct application of CRF or in vivo optogenetic activation of incoming CeA terminals, which constitute a major CRF input onto the LC, largely increases the tonic activity of LC neurons, with a subset of neurons decreasing activity in response to photostimulation.^6^^,^^79^ Similar in vivo results were established previously, with CRF increasing LC spontaneous firing following either local infusion into the LC or intracerebroventricular infusion.^80^, ^81^, ^82^, ^83^, ^84^ Importantly, sex differences in LC responses to CRF have been reported. CRF effects are exaggerated in females, as lower CRF doses can increase LC firing similar to what observed in males at higher concentrations.^41^^,^^85^ CRF-induced LC activation is associated with an enhanced release of NA and its metabolite (3-methoxy-4-hydroxyphenylglycol) in cortical and subcortical structures.^86^, ^87^, ^88^ Administration of CRF receptor antagonists alone has no effect on LC activity, indicating that CRF is not tonically released into the LC.^89^^,^^90^

Available evidence suggests that CRF mainly mediates its effects in the LC through CRFR-1, since application of a selective antagonist completely abolished CRF-induced increases in LC firing.^79^^,^^91^^,^^92^ Because no study has directly investigated the role of CRFR-2, the involvement of this receptor in the effect of CRF on the LC cannot be ruled out, particularly for longer-term changes that may lie further downstream of its signaling pathway. CRF binding to its receptor leads stimulates an adenylate cyclase-dependent signaling pathway.^93^ Although the detailed intracellular cascade induced beyond that is still incomplete, recordings performed in cultured LC cells demonstrate that CRF depolarizes the membrane through a cAMP-dependent reduction in potassium conductance.^79^ Other intracellular pathways are likely to be involved, and their recruitment appears to depend on sex and CRF concentration.^85^ Interestingly, the effect of CRF on tonic LC firing is attenuated by prior exposure to prolonged CRF administration or stress, suggesting a desensitization of CRFR-1.^80^^,^^94^ This desensitization may rely on the internalization of the receptor^74^ and is likely mediated by *β*-arrestin and Rab GTPase.^95^ This phenomenon is only observed in males, as prior exposure to an acute swim stress failed to modify LC response to CRF or induce internalization of CRFR-1 in females.^85^ Interestingly, there are interactions between CRFR-1 and other neuropeptide systems such that chronic exposure to the *μ-*OR agonist morphine appears to sensitize LC neurons to CRF.^96^ Furthermore, CRF can also mediate LC activity through modulation of glutamatergic inputs. Acute CRF application in slices increases the amplitude of spontaneous excitatory postsynaptic currents in a concentration-dependent manner.^97^ This effect seems to be restricted to a subset of inputs to the LC, notably coming from the periaqueductal gray (PAG).^98^

#### 3 Behavior

Direct infusion of CRF into the LC results in dose-dependent increases in stress-induced and anxiety-like behavioral responses without altering spontaneous motor activity.^99^ Conversely, infusion of *α*-helical CRF (*α*HCRF) 9-41, a competitive CRF receptor antagonist, into the LC leads to a dose-dependent reduction of foot-shock-induced freezing.^100^ CRF infusion into the LC has also been shown to improve cognitive flexibility, which could help promote adaptive behaviors during stressful situations. For example, low dose of CRF in the LC improves extradimensional set shifting, whereas a high dose of CRF facilitates reversal learning.^101^ Likewise, memory retention in a passive avoidance task was improved by either CRF injection into the LC or administration of yohimbine, which antagonizes autoinhibitory *α*2-adrenergic receptors. These results suggest that CRF enhances stress-related memories via activation of noradrenergic LC neurons.^102^

Activation of CRF^+^ projections from the CeA to the LC has similar stress- and anxiety-related behavioral effects. Photostimulation of CRF^+^ CeA terminals in the LC induces conditioned place aversion and decreases the amount of time spent in the open arms of the elevated zero maze (EZM), which assesses anxiety-related exploratory behaviors.^6^ Notably, the anxiogenic effect is reversed by infusing *α*HCRF into the LC prior to photostimulation and leads to an anxiolytic phenotype compared with *α*HCRF infusion in mice expressing enhanced yellow fluorescent protein instead of channelrhodopsin-2–enhanced yellow fluorescent protein, suggesting that the anxiety-like behavior induced by photostimulation is directly mediated by the release of CRF from CeA terminals in the LC.^6^ Systemic injection of antalarmin, a CRFR-1 antagonist, prior to photostimulation prevented the observed increase in anxiety-like behavior,^6^ further implicating CRFR-1 as the receptor upon which CRF acts to induce physiological and behavioral changes in the LC. CRF^+^ CeA terminals in the LC are also activated in rats that exhibit a short latency to defeat during a resident-intruder assay, both in response to a single exposure and to repeated stress.^103^

Pharmacological interactions between CRF and the NAergic system have been reported, which could reflect the role of CRF inputs to the LC in stress coping. Independent studies have demonstrated that systemic administration of yohimbine increases spontaneous burying behavior and attenuates CRF-induced increases in rearing and locomotion, whereas the *β*-adrenergic receptor antagonist propranolol potentiates CRF-induced hyperlocomotion and reduces CRF-dependent increases in burying, grooming, head shaking, and conditioned suppression of food reinforced behavior.^104^, ^105^, ^106^ Conversely, the *α*1-adrenergic receptor antagonist prazosin depresses behavior in a non–CRF-specific manner.^104^ Pretreatment with DSP-4 prior to systemic CRF administration blocked CRF-evoked burying but not grooming or head shaking behaviors.^106^ The same group later demonstrated that viral overexpression of CRF in Barrington’s nucleus, which increases CRF innervation in the LC, leads to increased burying behavior in a novel environment, with no effects on rearing or grooming.^107^ Diverging downstream targets from the LC may be responsible for modulating different coping behaviors; for instance, burying has been suggested to be regulated by LC projections to the forebrain.^106^ Together, these results indicate that CRF projections to the LC regulate various active stress responses, particularly burying behavior, via a *β*-adrenergic receptor-mediated mechanism.

### D Galanin

Galanin is a 29–30 amino acid neuropeptide originally isolated from the small intestine of pigs that has been implicated in feeding, pain, stress responses, depression, and substance use disorders.^108^, ^109^, ^110^ It is expressed in most (∼80%) rodent LC neurons and a smaller but still significant proportion of nonhuman primate and human LC neurons.^111^, ^112^, ^113^, ^114^, ^115^, ^116^ There are also galaninergic inputs to the LC originating from as yet unidentified sources.^117^

#### 1 Receptors and physiology

Galanin signals through 3 identified G protein-coupled receptors (GalR1, GalR2, and GalR3).^108^^,^^109^ GalR1 and GalR3 are typically coupled to G*α*_i/o_ proteins and inhibit cells, although GalR1 has been shown to switch to G*α*_s_ coupling under some conditions such as when forming—possibly transient—heterodimers with *μ*-ORs.^118^ By contrast, GalR2 is capable of coupling to multiple G proteins and mediates the persistent, neurotrophic properties of galanin. As a neuropeptide that is expressed in many LC neurons and potently suppresses LC firing, galanin is an obvious candidate for the predicted autocrine/paracrine activity of some LC-derived neuropeptides. However, the function of galanin is likely more complicated than it seems, providing useful examples of the challenges and opportunities that lurk in wait of future research on LC neuropeptide function.

Galanin is localized to and thought to be released from somatodendritic compartments of LC neurons.^118^^,^^119^ Galanin application to LC slices rapidly and potently suppresses LC neuron firing.^118^^,^^120^, ^121^, ^122^, ^123^ These effects appeared to be mediated by the G*α*_*i*/o_ coupled GalR1. The expression of GalR1 mRNA and protein in the LC was shown by autoradiography, in situ hybridization, RT-PCR, and immunohistochemistry.^124^, ^125^, ^126^, ^127^ Moreover, galanin-induced hyperpolarization of LC neurons in slice was mimicked by a mixed GalR1/GalR2 agonist but not a selective GalR2 agonist^121^ (Table 1) and was attenuated by small interfering RNA–mediated knockdown of GalR1 but not GalR2.^123^ Together, these data support a model by which somatodendritically released galanin acts via autocrine/paracrine signaling via GalR1 to provide negative feedback onto LC neurons, similar to what has been shown for the alpha-2 adrenergic autoreceptors. However, several methodological issues raise important limitations. For example, the antibody used to detect GalR1 protein was later found to be nonspecific, with no loss of signal when applied to tissue from GalR1 KO mice.^126^^,^^127^ In addition, the aforementioned autoradiography, in situ hybridization, and RT-PCR experiments were based solely on neuroanatomical landmarks and did not include a noradrenergic cell marker to verify colocalization.

Recently, more modern approaches to detecting receptor expression in a cell type–specific manner have further questioned these results, and therefore the model, suggesting that a revision of these concepts may be needed. Foster et al^128^ first noted a discrepancy using multilabel FISH to colocalize mRNA for galanin, GalR1, and TH in the LC of mice and rats. As previously reported, the authors found high levels of *Gal* expression in TH^+^ cells, but very low (∼19% of cells; ∼1 copy/cell) colocalization of *GalR1*. Instead, robust *GalR1* expression was detected in TH-lacking cells of the peri-LC, which was further confirmed at the protein level by mCherry immunohistochemistry in GalR1-mCherry knockin mice.^128^ These results conflicted with previous electrophysiology experiments which indicated that most LC neurons are inhibited by galanin^118^^,^^120^^,^^121^ (Table 1). The fleetingly low cell type–specific expression of GalR1 in noradrenergic LC neurons has been now repeatedly replicated, by TRAP, spatial transcriptomics, and snRNAseq in mice and/or humans.^29^^,^^37^^,^^39^^,^^44^^,^^45^

If GalR1 is not expressed in LC neurons, the model could be modified to predict an indirect circuit-based effect of somatodendritically released galanin on local non-noradrenergic GalR1-expressing neurons, which would then inhibit LC activity via a yet to be identified neurotransmitter or neuromodulator. Unfortunately, even this revised model does not fit some recent data. Using laser capture microdissection of human LC neurons based on the presence of neuromelanin, a pigment that accumulates exclusively in catecholamine neurons, RNAseq analysis indicated the presence of GalR1 mRNA in 13/20 subjects.^129^ Another study used single-cell RNAseq of mouse LC neurons and in situ hybridization to confirm GalR1 mRNA in GABAergic neurons surrounding the LC, but also found GalR1 mRNA at very low but detectable abundance (∼1–4 copies/cell) in ∼15% of TH^+^ neurons along the lateral edge of the LC.^28^ Importantly, accompanying slice electrophysiology experiments revealed that a preferential GalR1 agonist hyperpolarized ∼30% of LC-NA neurons that persisted in the presence of tetrodotoxine and picrotoxin, all but ruling out a circuit effect mediated by local GalR1-expressing GABAergic inputs. The authors attributed the discrepancy between the proportion of GalR1-expressing LC-NA neurons (15%) and the fraction of GalR1 agonist-sensitive cells (30%) to a bias in preferentially recording from the dorsomedial LC where the density of GalR1^+^/TH^+^ neurons is highest.

How can we reconcile all these findings? It could be that current neuroanatomical detection methods for GalR1 are not sensitive enough. It is also possible that only a few molecules of GalR1 protein engaged by agonists can powerfully inhibit LC activity. Galanin may even be acting at another receptor apart from GalR1, GalR2, and GalR3 that is more abundant in the LC. In addition, none of the experiments directly addressed whether endogenously released galanin from somatodendritic compartments has the same effect as bath-applied galanin receptor agonists in slice. What is clear is that conclusive determination of whether autocrine/paracrine signaling modulates LC activity and function for any given neuropeptide and receptor pair will require integrating a constellation of cell type– specific approaches, including mRNA/protein expression, gene KO, optogenetics, genetically encoded neuropeptide sensors, pharmacology, electrophysiology, and Ca^2+^ imaging.

#### 2 Behavior

LC-derived galanin has been implicated in many neuropsychiatric and neurological disorders including substance use disorder (particularly opioids), depression, anxiety, epilepsy, and Alzheimer’s disease.^109^ However, to our knowledge there are no reports of the consequences of intra-LC infusions on behavior, and only a few studies have used approaches that isolate the behavioral effects of LC-derived galanin, specifically. The first tool to examine this was a transgenic mouse that overexpressed murine galanin under control of the human dopamine *β*-hydroxylase (DBH) promoter. These mice display numerous phenotypes relevant to behavior including cognitive impairment,^130^ attenuated morphine withdrawal symptoms,^131^ increased voluntary ethanol consumption,^132^ resilience against the anxiogenic effects of foot-shock or optogenetic LC stimulation,^133^ and decreased seizure resistance.^134^ However, these results cannot be definitely assigned to LC-derived galanin because these mice overexpress galanin in all noradrenergic neurons, not just the LC, and also show ectopic expression of galanin in several non-noradrenergic nuclei in the brain.^135^^,^^136^ A complementary approach, noradrenergic-specific galanin KO, was achieved by crossing floxed galanin mice with DBH-cre animals, revealing increased marble and shock probe burying, as well as reduced latency to eat in the novelty-suppressed feeding (NSF) test,^117^ indicative of an anxiolytic effect. One caveat is that these mice lack galanin in all noradrenergic neurons, not just LC cells. Finally, because chronic exercise elevates galanin specifically in the LC,^109^ some properties of LC-derived galanin can be inferred by determining specific effects of exercise that can be prevented by galanin receptor antagonists. For example, the anxiolytic and anticonvulsant effects of exercise are attenuated by M-40, a nonselective galanin receptor antagonist.^137^^,^^138^ Moreover, the anxiolytic effects of exercise positively correlate with galanin expression in the LC.^117^

### E Neuropeptide S

Neuropeptide S (NPS) is a relatively newly identified peptide linked to arousal, with stress-relieving properties.^139^ NPS is expressed in a distinct population of TH-lacking neurons adjacent to the LC core,^140^ which are intermingled with—but mutually exclusive from—CRF neurons of the Barrington’s nucleus. Besides the peri-LC, NPS accumulates at the lateral parabrachial nucleus (PBN), where it resides within glutamatergic cells.^141^^,^^142^

#### 1 Anatomy

A recent study that employed rabies virus-based tracing of peri-LC NPS input-output relations demonstrated that these cells received input from variable sources, including several amygdalar and hypothalamic nuclei.^143^ Moreover, it was shown that peri-LC NPS neurons project heavily to the PVN, lateral hypothalamus, and the PAG validating earlier work based on immunohistochemical labeling.^144^ Currently, the functional role of incoming NPS input to the peri-LC as well as the postsynaptic targets of NPS-mediated neuromodulatory signaling in the region remain unexplored. Of note, peri-LC NPS^+^ cells coexpress galanin and NPY,^145^ arguing in favor of peptidergic coregulation of their targets. Conversely, these cells are found in close proximity to CRF^+^ fibers, and contain CRF-1 receptors,^146^ indicative of their regulation by peptidergic input. Indeed, CRF bath application leads to peri-LC NPS^+^ neuron depolarization and increased firing, effects mediated by CRF-1Rs, illustrating a direct interaction of the 2 peptides in the region. Besides *Crfr1*, peri-LC NPS^+^ neurons express mRNA for a plethora of relevant GPCRs, including the alpha-2A adrenergic receptor *(Adra2a), Npy1, Npy2r*, *Oprm1,* and *Oprk1.*^145^ Viewed together, (co)expression of NPS with other peptides and their cognate receptors further supports the idea of noradrenergic-neuropeptidergic feedback loops within the peri-LC space.

#### 2 Receptors and physiology

NPS receptors are detected throughout the rodent brain (eg, in the cortex, amygdala, PAG, dorsal raphe nucleus, and the PBN), but not the LC, with the highest levels seen in the thalamus and hypothalamus.^140^^,^^144^ In vitro studies have shown that nanomolar concentrations of NPS increases Ca^2+^ and cAMP.^139^ Assuming its corelease with glutamate, this would indicate a stimulatory effect of the peptide in neuronal physiology, possibly in concert with fast excitatory signaling. On the other hand, NPS application onto purified cortical synaptosomes inhibits depolarization-evoked NA release with remarkable specificity, as no effects in dopamine (DA) release were observed.^147^ The apparent discrepancies can be due to differential expression of NPS receptor subtypes in the model systems used. Independently, whether and how endogenous NPS signaling might affect LC-NA and neighboring peptidergic neuron physiology has yet to be determined.

#### 3 Behavior

As mentioned above, systemic NPS administration suppresses sleep, promoting wakefulness and has a general anxiolytic effect, seen in different anxiety-measuring tasks.^140^ Recent data suggest that peri-LC NPS^+^ neurons have minimal role in the sleep-disrupting properties of the peptide, with NPS signaling from the PBN being the main mediator of these effects.^143^ Stress exposure acutely engages peri-LC NPS^+^ neurons, as evident by increased c-Fos expression, supporting the notion that this population is probably involved in the anxiolytic effects of the peptide.^145^^,^^146^ The expression of CRF, NPY, and other arousal- and stress-related peptidergic receptors on these cells further strengthens this hypothesis. Finally, NPS signaling is involved in learning and memory processes as NPS KO mice show significant deficits in aversive and recognition memory consolidation and/or recall.^148^ Of note, in naïve mice, NPS administration enhances memory performance at the inhibitory avoidance task. These effects are blocked by pretreatment with the noradrenergic antagonist propranolol, indicating possible interactions between the 2 systems.^148^

As evident from the existing literature, the study of NPS-mediated signaling in the LC and peri-LC is at its infancy. Despite this, several lines of evidence point to functional interactions between NA, NPS, and other neuropeptides in the region. Future studies that employ more sophisticated methodologies can aid in dissecting the route and role of NPS neuromodulation of the LC-NA system as well as its behavioral implications.

### F Neuropeptide Y

NPY is a 36-amino acid peptide and among the most abundantly expressed peptides in the brain. Since the early 90s, the role of NPY in mediation of stress and anxiety has been consistently shown (reviewed by Heilig^149^ and Reichmann et al^150^), with NPY gaining popularity as the “stress-resilience” molecule^151^ following a series of preclinical studies highlighting its anxiety-relieving properties.^152^, ^153^, ^154^, ^155^, ^156^ The presence of NPY in the LC has been well established, with several early studies demonstrating its direct colocalization with LC noradrenergic neurons,^113^^,^^157^^,^^158^ and in close proximity to LC cell bodies and synapses,^67^ at least in the rat. More recent transcriptomic results taken from the mouse and human LC have determined that NPY expression is mostly limited to TH-independent neuronal clusters of the peri-LC region.^28^^,^^29^^,^^37^^,^^159^

#### 1 Anatomy

A few studies have examined the anatomical distribution of NPY afferents in the LC and peri-LC area. In rats, retrograde labeling targeting the LC revealed NPY-immunolabeled cell bodies in the hypothalamic arcuate nucleus.^160^ This sparse population (∼1.3% of arcuate NPY-expressing neurons) was only visible after concomitant colchicine injection, a methodological consideration when mapping peptidergic projections based on immunoreactivity. More recently, using viral-mediated retrograde tracing in NPY-Cre mice, it was shown that the LC receives distinct input from both local and distal sources.^159^ Indeed, NPY^+^ cell bodies were seen throughout the peri-LC, and several hypothalamic and pontine nuclei send extensive NPY^+^ inputs to the LC, with the strongest projectors (aside from local peri-LC afferents) including the lateral and posterior hypothalamic regions, the PAG, and the dorsal raphe nucleus. Of note, it was shown that more than half of LC-NPY axonal terminals coexpress CRF, but the source of this input has yet to be revealed.^67^ Given the opposing effects (inhibitory vs excitatory) of the 2 peptides on the LC, this surprising overlap indicates possible feedback mechanisms that drive LC-NA balance toward optimal firing rates. In addition, as NPY and CRF have contrasting behavioral roles (anxiolytic vs anxiogenic), coincident peptidergic release and dual modulation of the LC-NA system have important implications for adaptive behavior under challenging conditions.

#### 2 Receptors and physiology

NPY receptors are found on LC-NA neurons, as well as TH-lacking peri-LC neuronal populations, with NPY receptors Y1R and Y2R being the most abundant in the region.^67^^,^^161^, ^162^, ^163^ However, as mentioned above, discrepancies in the extent of NPY receptor localization have been reported. Although *Npy1r*, *Npy2r*, and *Np5r* mRNA was detected in LC-NA neurons by single-cell sequencing,^28^^,^^29^ one study did not confirm their NA localization by in situ hybridization.^28^ Others have shown that both Y1Rs and Y2Rs are present in the LC, with Y1R being preferentially expressed in postsynaptic dendritic terminals and Y2R in presynaptic axonal terminals.^67^ Single foot-shock exposure causes a persistent increase in *Npy1r* and *Npyr2* expression in the LC, implicating NPY-mediated signaling in the mediation of stress responses.^164^ Of note, as with NPY itself, receptor expression often occurs in LC-projecting CRF terminals,^67^ further indicating possible complex interactions between these 2 neuropeptidergic LC modulators.

Recently, it was shown that the majority of LC-NA neurons (∼90%) contain mRNA for either *Npy1r Npy2r* or both and receptors,^159^ a finding that is supported by the well documented effects of NPY on LC-NA physiology (Table 1). In particular, exogenously applied NPY reduces LC-NA neuron spontaneous discharge^165^ and facilitates their hyperpolarization. The latter effect was mediated by Y2Rs, as it was replicated by application of the Y2R/Y5R agonist NPY13-36 and not the high-affinity Y1R agonist [Leu^31^,Pro^34^]-NPY.^166^^,^^167^ Notably, bath-applied high and low NPY concentrations trigger opposing effects on noradrenergic cell excitability, respectively, increasing or decreasing NA firing rates.^159^ These NPY effects are distinctly mediated by the different YRs present in the region. Particularly, selective Y1R antagonism blocks low NPY-induced decrease in LC-NA excitability, indicating direct actions of NPY on LC Y1Rs. Conversely, LC-NA hyperexcitability following high NPY concentrations was abolished by either a selective Y2R antagonist or a cocktail of blockers for glutamatergic and GABAergic synaptic inputs. Together these results support direct and indirect network effects of NPY on LC physiology. Beyond the effects of pharmacologically applied NPY, it was recently demonstrated that endogenous NPY triggers LC activity-dampening effects.^159^ In particular, it was shown that chemogenetically evoked NPY release from the local, peri-LC NPY population hyperpolarizes LC-NA cells and reduces their excitability, an effect mediated by postsynaptic Y1Rs. Together, available anatomical and physiological data from mice and humans indicate that NPY belongs to a type 2 neuromodulator of the LC, mostly absent from NA neurons themselves but able to regulate their activity via its receptors (Fig. 3).

#### 3 Behavior

There is limited data establishing NPY behavioral effects due to direct interaction with the LC-NA system. An early study showed that microinjection of NPY or NPY13-36 into the peri-LC region of the rat results in anxiolytic effects in the elevated plus maze (EPM), as seen in increased exploration and entries into the open arm.^168^ Likewise, the same group showed that intracerebroventricular administration of NPY prevents social interaction deficits following noradrenergic lesions by systemic pretreatment with DSP-4.^169^ The above-mentioned work used exogenously applied NPY to examine its neuromodulatory effects on LC and subsequent regulation of behavior. Recently, a study employing intersectional manipulations of peri-LC NPY cells showed that endogenous chemogenetically evoked NPY release in the LC reduces baseline anxiety-related behaviors in the EPM and the NSF tasks, an effect that is mediated by Y1Rs. Conversely, peri-LC NPY inhibition increased latency to feed, and reduced food consumption at the NSF, both indicative of anxiogenesis when local NPY signaling is abolished.^159^ These latter findings indicate that NPY exerts strong neuromodulatory control over the LC to drive bidirectional changes in anxiety-like behavior.

In addition to studying naïve animals, a few studies have also examined NPY behavioral effects following stress. In rats previously exposed to a combination of physical stressors, single NPY intranasal administration sufficiently prevented stress-induced anxiogenesis,^170^ an effect that is in parallel with restoration of stress-induced changes in LC TH protein and *Npy2r* mRNA expression.^171^ Albeit indirectly, these results indicate that NPY’s stress-reducing properties are at least partially mediated by the LC. Definitive support for this concept comes from recent findings demonstrating that chemogenetic activation of peri-LC NPY-expressing neurons prevents foot-shock-induced reduction in time exploring the open arms of an EPM, indicating anxiolysis.^159^

Collectively, available data highlight local NPY-mediated neuromodulation of the LC underlying the peptide’s anxiety-relieving properties, under both naïve and stressed conditions. As we continue to employ novel intersectional methods for the identification and selective manipulation of distal LC NPY inputs, it will be intriguing to determine whether NPY contributes in a region-specific manner to the regulation of distinct behaviors.

### G Opioids

ORs were first discovered by Pert and Snyder^172^ in 1973 and were identified as a target in the LC just a few short years later.^173^ In the intervening years, much more information regarding the types of ORs (*μ*-, *κ*-, *δ*-, and nociceptin), their signaling properties, and their behavioral implications has been established. Although often thought to counter the stress responsivity of LC neurons, LC ORs also appear to have roles typically associated with ORs elsewhere such as pain control, reward, and aversive processing.

#### 1 Anatomy

The LC is regulated by multiple endogenous opioids (each with several cleavage products), primarily ENK and DYN. Endogenous ENK input to the LC, primarily the LC core, comes from several brainstem sources, including the prepositus hypoglossi, the nucleus paragigantocellularis, and the rostral medulla.^174^, ^175^, ^176^ More than 50% of these LC-projecting brainstem neurons are immunoreactive for ENK,^173^ which extends to the peri-LC dendritic zones more rostrally.^177^^,^^178^ By contrast, DYN innervation of the LC arises from the CeA and highly overlaps with CRF, with little colocalization of ENK.^69^ This convergent pattern of CRF/DYN and ENK innervation from distal sources has served as a substrate for the instantiation of and recovery from stress.^179^^,^^180^ Recent work using either Cre-dependent reporter lines^181^^,^^182^ or intersectional targeting strategies^182^ has identified a subpopulation local LC-NA neurons that also express DYN. A complete anatomical map, however, of these type 1 LC-DYN neurons has yet to be established.

#### 2 Receptors and physiology

Mirroring their pattern of afferent input, there is abundant expression of a variety of ORs in and around the LC. These include the *κ*-, *μ*-, *δ*-, and nociceptin ORs. Although *μ*-ORs are largely expressed in LC-NA neurons,^174^^,^^183^ *κ*-ORs appear to primarily mediate presynaptic inhibition of inputs to the LC.^184^ Expression of *δ*-ORs within and surrounding the LC is less well characterized but has been shown to more closely resemble *κ*-OR expression patterns with expression primarily on presynaptic inputs.^185^ Further contrasting with *μ*-ORs, which are found on the plasma membrane of LC noradrenergic neurons,^178^ *δ*-ORs are expressed intracellularly on dense core vesicles.^185^ Given that endogenous ENKs (Met-ENK and Leu-ENK) have higher affinity for *δ*-ORs than *μ*-ORs,^186^ more thorough characterization of cellular and subcellular *δ*-OR expression patterns within the LC is warranted. Finally, the LC contains populations of both nociceptin opioid peptide (N/OFQ)-synthesizing and nociceptin opioid peptide receptor (NOPR)–expressing neurons, though their precise expression patterns and behavioral function are largely unexplored.^29^

The electrophysiological responses and intracellular signaling cascades initiated by activation of ORs in LC-NA neurons have long been studied using a diverse set of pharmacological agents in both ex vivo and in vivo conditions (Table 1). Among the 4 classical ORs, the *μ*-OR has been the focus of the most in-depth studies. LC-NA neurons robustly express *μ*-OR, which has been carefully documented through morphological, electrophysiological, and genetic evidence.^37^^,^^39^^,^^178^ Despite presumably lacking direct endogenous opioid peptides in the LC under normal slice electrophysiological conditions,^187^^,^^188^ the acute application of *μ*-OR agonists inhibits adenylyl cyclase activity by phosphorylating the G_i/o_ subunit, resulting in a reduction of cAMP production.^189^, ^190^, ^191^ The G*βγ* subunits inhibit calcium channels and cause a robust suppression of spiking through G protein-gated inwardly rectifying potassium channel (GIRK) subunits 2/3–mediated hyperpolarized current.^192^, ^193^, ^194^, ^195^ Furthermore, G-protein-independent *β*-arrestin signaling recruits GRK2/3-mediated phosphorylation of the *μ*-OR, further promoting desensitization and internalization of the receptor itself.^196^, ^197^, ^198^, ^199^, ^200^, ^201^ Beyond acute application, withdrawal following chronic exposure to *μ*-OR agonists induce hyperactivity in LC-norepinephrine (NA) neurons. This long-term adaptation arises with opioid tolerance and has been thoroughly studied at the behavioral and cellular levels.^202^, ^203^, ^204^ This state is characterized by upregulated cAMP, protein kinase A, and cAMP response element-binding protein activity that augments LC activity and opiate dependency that is unmasked by naloxone-precipitated withdrawal after prolonged morphine exposure.^205^, ^206^, ^207^, ^208^, ^209^, ^210^ As previously noted, long-term morphine exposure sensitizes responses to CRF and physiological stressors.^96^ To our knowledge, this is the only study reporting the effect of ongoing chronic *μ*-OR agonist on LC firing without later *μ*-OR antagonism and remarkably suggests a decrease in baseline discharge after 7 days of morphine exposure. Further study is merited on the effects of LC firing following chronic opioid exposure in absence of *μ*-OR antagonism.

The other ORs have received less attention in the context of the LC-NA system. With regard to *κ*-OR, it has been demonstrated that local infusions of *κ*-OR agonists attenuate phasic activity evoked by sensory stimuli in LC.^184^ Results from ex vivo electrophysiology studies also show evidence of presynaptic modulation targeting synaptic input onto LC-NA neurons from selective afferents during the activation of *κ*-ORs and *δ*-ORs.^98^^,^^211^, ^212^, ^213^ Overexpression of *δ*-ORs in the LC alleviates stress-induced increase in spontaneous firing rates of LC-NA neurons.^214^ Along similar lines, chronic antagonism of *κ*-ORs decreases phosphorylated extracellular signal-regulated kinase, a marker of intracellular signaling and neural activation, suggesting an ongoing dynorphinergic tone in the LC.^215^ Finally, as noted earlier, recent work has identified a subset of LC-NA neurons express prodynorphin (Pdyn), a precursor protein of DYN, leading to a possible scenario of *κ*-OR-mediated regulation through the release of DYNs.^182^ These recently identified LC NA^+^/DYN^+^ neurons may represent a type 1 (Fig. 3), where their neuropeptide release may lead to presynaptic modulation of inputs to themselves, other LC-NA neurons or the feedback to the peri-LC.^98^ Based on limited expression of *κ*-OR on LC-NA cells, the peri-LC scenario seems more plausible.

As the least studied member of the OR family, the full intracellular response of NOPR activation in LC has yet to be revealed. Like *μ*-OR, it is well known that the LC has enriched expression of NOPR.^29^^,^^37^ Also similar to *μ*-OR, the application of nociceptin robustly suppresses action potentials through GIRK as well as reducing calcium conductance.^216^, ^217^, ^218^, ^219^, ^220^ More efforts are needed to clearly reveal the intracellular signaling of NOPR in the LC, as well as the physiological and behavioral effects of these abundantly expressed LC receptors.

#### 3 Behavior

Given the wide variety of OR expression within the LC, effects of opioid administration on behavior are mixed. Consistent with *μ*-OR expression patterns in LC-NA neurons, early experiments reported that lesioning the LC dramatically reduces morphine-induced analgesia^221^^,^^222^ whereas stimulation of the LC produces or enhances analgesia.^223^, ^224^, ^225^ In rats, intra-LC infusion of met-ENK produces dose-dependent analgesia as measured by the tail-flick test, which rapidly produces tolerance after 3 days of administration.^226^ Similarly intra-LC infusion of the highly selective *μ*-OR agonist [D-Ala^2^, N-Me-Phe^4^, Gly^5^-ol]-ENK produces antinociception to a thermal stimulus, which is reduced during inflammatory pain, possibly due to a downregulation of *μ*-ORs in the LC.^227^ Recently, it was shown that restoration of *μ*-OR expression selectively in LC-NA neurons following neuropathic injury reverses mechanical hypersensitivity.^219^ This latter effect on antinociception and analgesia is thought to be mediated by LC neurons projecting to the prefrontal cortices.^219^^,^^220^ Exogenous activation of *μ*-ORs also recruits descending LC projections in contribution to morphine analgesia.^228^ Consistent with its potential opposing influence on stress, intra-LC ENK infusions reduce stress-induced anxiety-like behavior and produce conditioned place preference. Interestingly, stress was associated with a reduction in LC *δ*-OR expression (though expression of *μ*-OR was not examined), and overexpression of *δ*-OR using viral vectors driven by a synthetic DBH promoter (PRSx8) for LC specificity prevented stress-induced anxiety-like behavior.^214^ In the peri-LC, chemogenetic stimulation of ENK- or nociceptin-expressing neurons both decrease the amount of time mice spend in the open arms of the EZM, suggesting that local opioidergic LC inputs promote anxiety-like behavior,^29^ possibly via disinhibition of LC-NA neurons. Future experiments manipulating ENK inputs to LC may further tease apart the role of endogenous opioid signaling in pain- and stress-related behaviors.

As discussed above, there is a large degree of overlap between CeA CRF-expressing and DYN-expressing projections to LC^229^; thus, some of the stress-related behavioral regulation attributed to activation of CeA projections to LC may in part be mediated by the *κ*-OR.^6^ Microinfusion of the long-acting *κ*-OR antagonist norbinaltorphimine^230^ into the LC prevented reinstatement of cocaine place preference elicited by the *κ*-OR agonist U50,488.^215^ This same effect was recapitulated with LC-specific lentiviral overexpression of *κ*-OR and was prevented by blockade of *β*-adrenergic receptor signaling, indicating an interaction between the DYN/*κ*-OR and adrenergic systems.^215^ Furthermore, activation of the recently identified local LC-DYN neurons drives an anxiogenic state in mice.^182^ Although very little is known about the in vivo role of NOPR in the LC, emerging evidence suggests it is not necessary for LC-mediated baseline control of nociception.^219^ Much more work will be necessary to have a clearer understanding of the behavior function of *δ*-OR and NOPR in the LC.

### H Orexin/hypocretin

Orexin A (ORXA) and orexin B (ORXB), also referred to as hypocretins 1 and 2 (HCRT1/2), are cleaved from the prepro-orexin precursor, encoded by the *Hcrt* gene. Orexins are exclusively synthetized by neurons located in the lateral hypothalamus^231^^,^^232^ and are involved in physiological functions such as wakefulness, arousal, and energy homeostasis. Aside from intrahypothalamic connections, the LC represents the main efferent target of the orexinergic system,^232^, ^233^, ^234^ where it regulates attention, sleep-wake cycles, and locomotion.^235^

#### 1 Receptors and physiology

Orexins act through 2 distinct receptors: orexin A receptor (ORXA-R) and ORXB receptor. Although ORXA and B bind to ORXB receptor with the same affinity, ORXA possesses a 10-fold higher affinity for ORXA-R than ORXB.^236^ Orexin receptors are usually G_i/o_ or G_q_-coupled^237^, ^238^, ^239^ and therefore inhibit adenylyl cyclase or activate phospholipase C, respectively. Some evidence suggests the possibility of G_s_ coupling, leading to an increase in adenylyl cyclase activity.^238^ Both orexin receptors are expressed in the LC, with ORXA-R being particularly abundant^240^^,^^241^ and available evidence suggests that orexins excite the LC (Table 1). Application of both ORXA and ORXB increases LC firing frequency^220^^,^^242^^,^^243^ in vitro, with ORXA having a bigger effect than ORXB on LC firing,^244^ consistent with ORX-R expression in the LC and the enhanced affinity of ORXA for its receptor.^236^ The orexin-induced excitation is most likely mediated by decreased potassium conductance^245^ and suppression of GIRK channel activity.^246^ In addition, ORXA decreases inhibitory transmission onto the LC, through an ORXA-R/cannabinoid type-1 receptor–dependent mechanism.^247^ Similar effects are observed in vivo, as intra-LC intracerebroventricular orexin delivery increases LC firing and NA release in the prefrontal cortex and dentate gyrus.^248^^,^^249^ Accordingly, photostimulation of orexin terminals within the LC triggers LC-NA depolarization and fast excitatory synaptic responses, in an AMPA- and ORXA-R-dependent manner.^250^

#### 2 Behavior

In accordance with the putative role of orexins in behavior, and the robust effects of orexin signaling within the LC, orexin-NA interplay is observed in a large variety of behavioral paradigms. First, ORXA-R-mediated LC depolarization and enhanced firing is in parallel with increased arousal and locomotion.^251^ Then, LC-NA ablation attenuates the sleep-promoting effects of orexin antagonism, indicating a direct interaction of the 2 systems in sleep-wake cycles.^252^ In support, photoinhibition of the LC prevents sleep-to-wake transitions driven by optogenetic activation of hypothalamic orexin neurons, and vice-versa, LC photostimulation enhances orexin effects on wakefulness.^253^

Besides arousal, orexin enhances aversive learning via its effects in the LC. In particular, selective reinsertion of ORXA-Rs in LC-NA neurons normalizes impaired fear learning in ORXA-R global KO mice.^254^ Likewise, mice lacking the ORXA-R specifically in noradrenergic neurons show decreased freezing after conditioning, further illustrating the necessity of orexin-mediated modulation of the LC in learning.^255^ Furthermore, photostimulation of LC orexin terminals facilitates learning, as seen in increased freezing levels, an effect blocked by ORXA-R antagonism.^250^ Notably, pharmacological disconnection of the hypothalamic orexin-to-LC and LC-to-amygdala pathways mimics this behavioral effect, mapping the neuromodulatory circuits underlying fear learning.^250^ Of note, these results were replicated more recently using intersectional approaches, where photoinhibition of monosynaptic, hypothalamus-LC orexin signaling decreased fear expression. Conversely, it was shown that photostimulation of this pathway evokes fear responses in nonthreatening environments.^255^ Taken together, orexin-mediated modulation of the LC is crucial for an extensive behavioral repertoire that includes arousal/awareness, attention, learning, and memory.

### I Somatostatin

Somatostatin (SST) was first identified as a growth hormone inhibitor.^256^ The cyclic peptide is found throughout the brain in 2 major isoforms, the 14 amino acid and a 28 amino acid version each arise from the same prohormone.^257^ Its role in many neuropsychiatric disorders has been reviewed recently,^258^ but its role in the LC is relatively understudied. SST-expressing pericoerulean neurons are located exclusively at the ventromedial tip of the LC proper and are distinct from the local NPY^+^ population.^159^ Furthermore, the LC expresses mRNA for at least 3 of the 5 subtypes of SST receptors: *Sstr1*, *Sstr2*, and *Sstr3*.^29^^,^^37^^,^^259^

All studies investigating the functional effect of SST on LC activity have used acute ex vivo slices and consistently report hyperpolarization of LC neurons along with a decrease in firing rate (Table 1). Notably, this effect involves the activation of inwardly rectifying K^+^ conductance in LC neurons^260^ and possibly a pertussis toxin-sensitive GTP-binding protein.^261^ SST mechanism of action in the LC seems to be independent from the inhibition of cAMP formation and mainly mediated by the SSTR2.^262^ The sole behavioral investigation suggests that local infusion of an SSTR antagonist into the LC reduces rapid eye movement sleep, suggesting a potential endogenous role of the system in arousal and sleep dynamics. Given the inhibition elicited by SST application onto LC-NA neurons, further study is warranted to determine whether this system functions like other LC inhibitors in stress, pain, and opioid withdrawal.

## III Tools and advances in studying neuropeptidergic-driven modulation of the locus coeruleus

Given the robust evidence of neuropeptide and receptor expression within the LC, further study of these multifunctional cells requires knowledge of when each neuropeptide arrives to or is released from LC-NA neurons. Neuropeptide signaling can be monitored in a number of ways, and several recently published reviews for in-depth discussion of these methods are available.^263^, ^264^, ^265^ Here, we briefly describe some of these methods and highlight their relevance to the study of neuropeptides in the LC-NA system.

### A Intersectional targeting of neuropeptidergic locus coeruleus cells

The study of neuropeptide-expressing LC-NA neurons necessarily requires targeting these cells at the intersection of NA and neuropeptide production. Intersectional targeting is commonly accomplished using transgenic mouse lines expressing orthogonal recombinases (eg, Cre and Flp) driven by genes expressed in the target cell population (eg, *Dbh* and the neuropeptide of interest). Dual recombinase-expressing mice can then be crossed with a double-recombinase-dependent gene of interest to drive expression solely in cells containing both recombinases.^266^, ^267^, ^268^ These transgenic mice offer access to these cellular populations and are particularly useful in labeling cells throughout development, in sparse and difficult-to-access populations, and in situations requiring bulky transgenes. However, transgenic mice lack temporal and regional specificity since the intersectional transgene will be expressed in any cell containing both recombinases at any point throughout the animal’s development. Thus, it cannot be assumed that only the population of LC cells of interest will be targeted. Therefore, several intersectional adeno-associated viral strategies have been developed,^182^^,^^269^, ^270^, ^271^, ^272^, ^273^ each with their unique benefits and drawbacks. Although the packaging capacity of adeno-associated viral vectors limits the size of transgene able to be delivered, they offer spatial and temporal resolution that is lost with intersectional transgenic mice. With these targeting strategies, peptidergic LC cells have been successfully targeted to investigate their effects on behavior. For instance, conditional viral expression by ribozyme guided degradation (ConVERGD)-based rabies tracing and excitatory designer receptors exclusively activated by designer drugs have been used to investigate the circuitry and behavioral effects of DYN-expressing LC NA^+^ (or DBH^+^) cells.^182^

### B Neuropeptide and noradrenaline detection

#### 1 Fluorescent calcium indicators

Aside from tracing the anatomical connections and manipulating the function of neuropeptide-expressing LC cells, intersectional strategies can also be used to monitor the activity of these subpopulations. Expressing genetically encoded calcium indicators like GCaMP in these cells enables the selective recording of activity through fiber photometry, microendoscopy, and 2-photon imaging, each of which has its pros and cons. For instance, fiber photometry is the least invasive recording method and can be a good choice for recording the bulk activity of sparse populations of cells (like peptidergic LC cells), but it lacks the cellular resolution obtained with microendoscopy or 2-photon imaging. This limitation is particularly problematic when considering the emerging view of functional heterogeneity within the LC,^27^ but can be overcome to an extent with retrograde approaches. Microendoscopy allows cellular resolution in freely behaving animals, but the surgeries involve implantation of relatively large lenses which can be damaging when imaging deep brain regions. Two-photon imaging offers the greatest spatial resolution and could be a good choice for studying populations with a low signal to noise ratio, but it almost always requires mice to be fixed to a head-post which eliminates recording in freely moving settings.

Calcium indicators have been widely used to study the activity of the LC in the context of NA signaling.^274^ Recent work leveraged a fast and bright genetically encoded calcium indicator^275^ to produce a higher throughput physiological assay for quickly assessing GPCR- and neuropeptide-mediated changes in LC-NA firing rates based on their efferent projection profiles.^220^ It is worth noting that LC-NA neurons have rather unique calcium handling properties that give rise to tonic action potentials even in the presence of voltage-gated sodium channel blockers.^16^^,^^276^ However, given the dual-effector nature of neuropeptide-expressing LC neurons, the activity of these cells cannot be assumed to solely trigger NA release. To better understand the identity of signaling molecules released from these cells, one must also use detection methods capable of sensing coincident neuropeptides.

#### 2 Neuropeptide biosensors

Similar to calcium indicators, which detect fluctuations of intracellular calcium as a proxy for cellular activity, many groups have developed fluorescent indicators which are specific to different signaling molecules. Although NA-based sensors are certainly relevant for studying LC-related signaling (see the study by Tanguay et al^274^ for a detailed review), the focus here is instead on peptide-specific indicators, in line with the goal of this review to highlight neuropeptide release within and from the LC. Recent advancements in fluorescent neuropeptide sensors have expanded detection to a broad array of neuropeptides including *β*-endorphin, DYN, met-ENK, SST, nociceptin, CRF, cholecystokinin, NPY, neurotensin, vasoactive intestinal peptide, oxytocin, orexin, and glucagon-like peptide.^277^, ^278^, ^279^, ^280^, ^281^, ^282^ Although development of these sensors is rapidly improving, in some cases they still lack the ability to detect and differentiate between more similarly structured neuropeptides (eg, endogenous opioids), primarily due to the imperfect binding specificity of the GPCRs on which these sensors are typically based. Furthermore, the low physiological concentrations at which some neuropeptides reside may be insufficient to generate substantial signal to noise ratios for reliable detection by many of these sensors, even with sensitive recording methods like 2-photon imaging. Due to these issues, use of in vivo peptide KO approaches, in combination with pharmacological validation experiments, is necessary for clear interpretation of biosensor-based experiments. Indeed, combining pharmacological and genetic perturbations with advancements in biosensor specificity and fluorescence detection have already proven useful for the investigation of protein dynamics in the mouse brain,^283^ paving the way for future studies into the peptidergic dynamics of the LC system.

#### 3 Fast-scanning cyclic voltammetry

Fast-scanning cyclic voltammetry (FSCV) is an electrochemical method used to detect the presence of neurotransmitters, metabolites, and even neuropeptides. By rapidly cycling the voltage of a microelectrode between a resting and peak voltage, molecules near the electrode are repeatedly oxidized and reduced resulting in a measurable current. Since all molecules have a unique redox potential, high levels of specificity can be achieved by tailoring the electrode composition and input voltage waveform to target the molecular signature of the biomolecule of interest. In some cases, this high level of specificity can even be expanded to the simultaneous detection of multiple molecules in vitro.^284^ Historically, FSCV was used to detect small molecules, but recent advances in probe construction and waveform administration have expanded specific detection to neuropeptides as well.^285^, ^286^, ^287^ However, despite its theoretical specificity and proven efficacy with many small molecule neurotransmitters, establishing effective FSCV protocols for new molecules is not a simple task, especially when attempting to distinguish between neurotransmitters with similar redox potentials, such as NA and DA,^285^ or larger molecules like neuropeptides, many of which have a similar molecular backbone. Additionally, since FSCV can only detect biomolecules in the direct vicinity of the probe, the low physiological concentrations of some neuropeptides may be beyond the detection range of current probes. Despite these caveats, FSCV is nevertheless a beneficial method for recording the rapid changes in signaling molecules. Given its small probe size, it also allows for the relatively easy placement in deep brain areas, making it a useful tool for studying LC circuit dynamics. Indeed, FSCV has been paired with pharmacological manipulations to gain insight into the details of NA signaling from the LC^288^, ^289^, ^290^ even to the effect of differentiating between NA and DA fluctuations upon LC activation.^291^^,^^292^ FSCV has also been paired with more modern circuit-manipulation techniques such as optogenetics to detect downstream signaling caused by LC activation.^293^, ^294^, ^295^ FSCV remains an important tool for measuring precise signaling dynamics, and the further development of neuropeptide-specific detection in combination with improved circuit-manipulation techniques will surely help to progress the field’s knowledge of peptidergic LC circuitry.

#### 4 Microdialysis

Microdialysis involves implanting a small semi-permeable membrane catheter through which extracellular fluid is collected. These samples can then be analyzed by a variety of chemical detection strategies such as radioimmune assays, ELISA, and tandem liquid chromatography- mass spectrometry to determine the presence of biomolecules including neurotransmitters and neuropeptides. This combination of analytical methods is very sensitive and can detect nearly all molecules. Microdialysis is a great tool for detecting low concentrations of biomolecules, such as neuropeptides released from small populations of LC cells. However, the sample collection occurs over large time periods dependent on the flow rate of the dialysis system, so this method is not ideal for observing quick changes in signaling molecules.^263^, ^264^, ^265^ For this reason, microdialysis is often combined with detection methods with finer temporal resolution such as FSCV which together can detect very small, dynamic changes in neuropeptides like ENKs and DYNs for which until recently there have been no fluorescent sensors available.^296^^,^^297^ Additionally, microdialysis remains the gold standard for neurotransmitter and neuropeptide detection, due to its ability to quantify absolute amounts of a given molecule within a sample as opposed to other detection methods that can only measure changes from baseline. As discussed in the next section, microdialysis and its subsequent analytical approaches also maintain some level of clinical translation as it can be deployed on patient samples relatively easily.

#### 5 Functional imaging

Although invaluable insights have been gleaned from the detection methods mentioned above, their invasive nature largely precludes their ability to easily transition to clinical studies with human subjects. For that reason, clinically relevant detection methods like functional magnetic resonance imaging (fMRI) and positron emission tomography (PET) may be preferable for translational studies. fMRI typically measures changes in blood oxygenation as a proxy for neural activity. However, advances in heme-based contrast agents have allowed the specific detection of neurotransmitters like DA.^298^ Development of similar contrast agents that are selective for neuropeptides would allow the brain-wide fMRI detection of neuropeptides. PET measures the degradation of supplemented radioligands. The aggregation of these tracers serves as a proxy for where the ligands are used in the brain. This method allows the visualization of endogenous receptors,^299^ but it does not allow for the visualization of endogenous release, which would be crucial for studying neuropeptide-expressing LC cells. Functional imaging methods, while clinically relevant, have some drawbacks, particularly when studying small neural populations like peptidergic LC neurons. These methods require expensive detection machinery leading to large start-up costs which could prohibit their use in basic research studies. Additionally, they lack the cellular and temporal resolution that would be necessary for investigating small populations of LC cells. Still, MRI and PET imaging have both been used to investigate the LC,^300^, ^301^, ^302^, ^303^, ^304^, ^305^, ^306^ though not in the context of neuropeptide signaling.

## IV Noradrenaline-peptide cotransmission

The simultaneous existence of multiple small molecule (eg, NA, DA, and glutamate) and neuropeptide (see above) neurotransmitters in individual LC neurons may offer the possibility that these neurons corelease multiple transmitters. Indeed, given the high degree of signaling molecule coexpression in the LC, the number of potential combinations is staggering. At this time, very little is known about cotransmission in the LC. On the basis of mRNA or protein coexpression alone, it is challenging to draw a definitive conclusion on whether NA and LC neuropeptides are coreleased or do merely co-occur. Indeed, several fundamental questions remain, including whether these molecules are delivered by the same release sites; whether they are stored individually or together in vesicles; under what conditions they are released; and how their signals are integrated and interpreted by the target cell. Fortunately, recent advances in neuroscience tools and technique development have primed the feasibility of directly asking these questions,^307^ and there is now a handful of studies whereby corelease has been established.^308^^,^^309^ For example, Yang et al^310^ presented compelling evidence for NA-glutamate corelease in the PBN, which both excites PBN neurons and relieves an amygdala-originated inhibitory brake to the region, with implications for (fear-evoked) feeding behaviors.

To our knowledge, only one study has directly examined the consequences of peptidergic cotransmission from LC neurons, in this case NA and galanin. Stress-induced anxiety was assessed in mice specifically lacking NA but expressing normal LC galanin levels (DBH KO) and in mice specifically lacking noradrenergic-derived galanin but showing normal LC-NA levels (GAL^cKO-Dbh^).^311^ It was found that foot-shock stress produced persistent (24 hours) anxiety-like behavior in wild-type mice, an effect absent in both DBH KO and GAL^cKO-Dbh^ mice. Restoring NA at the time of the anxiety test, but not the time of the stressor, rescued anxiety-like behavior. Conversely, restoring galanin signaling at the time of the stressor, but not the time of the anxiety test, 24 hours later, rescued anxiety-like behavior.^311^ Furthermore, pharmacological rescue of NA, but not galanin, in KO mice during EZM testing was anxiogenic. Consistent with an earlier report that targeting LC-GAL neurons recapitulates the real-time aversive effects of LC photostimulation,^6^ indicating that NA and NA-derived galanin play complementary, but distinguishable roles in behavioral responses to stress. Based on these results, NA is required for the expression of acute stress-induced anxiety, whereas noradrenergic-derived galanin mediates the development of more persistent responses following a stressor. The LC represents an ideal neuronal population for future fundamental neuroscience research dissecting how cotransmission of small molecule neurotransmitters and neuropeptides, or of multiple neuropeptides, influences physiology and behavior.

## V Conclusions

In the last decade, we have witnessed a resurgence of interest in the LC-NA system,^3^ driven by technological advances that enabled a more nuanced analysis of the LC molecular identity and functional neuroanatomy. It is now widely accepted that LC-NA cell ensembles can be differentiated based on their transcriptional profile,^28^^,^^29^^,^^37^ afferent input,^98^ and projection patterns.^7^^,^^312^ Here, we discussed the state of the field with regard to neuropeptidergic signaling in the LC, outlining a rich source of heterogeneity within the region. Indeed, by integrating coexpressed peptides with their cognate receptors, NA cells can be classified more distinctly, with tangible functional implications for the LC’s neuromodulatory influence throughout the brain.

As mentioned above, due to binding multiple and various GPCRs across brain areas, and the expansive nature of neuromodulatory signaling (eg, volume-transmitted), potential NA/peptidergic corelease enables dynamic gain control across diverse temporal scales and amplitudes. This multiplexed control likely arises from synaptic, cellular, and network-level effects. As we continue to decode the principles underlying NA/peptide cosignaling, including release and reuptake dynamics, we will be more aptly equipped to understand how these GPCRs refine the far-reaching effects of LC-mediated neuromodulation.

Alongside their ability for peptidergic transmission, and possibly cotransmission, LC neuronal clusters are transcriptionally defined by the presence of cognate GPCRs, rendering them selectively responsive to peptide-mediated neuromodulation. Peptidergic signaling can dynamically alter LC-NA neuronal excitability and discharge rates.^6^^,^^28^^,^^159^ LC firing rate variability, and the associated capacity for phasic versus tonic NA release, strongly determines the engagement of projection regions and is therefore a key component of the consequent behavioral outcomes.^293^^,^^313^^,^^314^ It is then plausible that the dominant peptidergic afferent drive onto LC-NA neurons could act to critically shape the mode and spatiotemporal aspects of LC firing patterns, to ensure optimal information relay and adaptive behavioral responses to internal and external demands.

Taken together, peptidergic neuromodulation of the LC could sustain polymodal and heterogeneous NA neuronal subsets, regulated in a peptide input-specific and receptor-dependent manner, supporting LC modularity of function. Given the broad range of LC projections, it has been suggested that the LC can switch between functioning discretely or contributing to brain-wide state shifts in a context-specific manner to more precisely coordinate adaptive behavioral responses.^315^ By fine-tuning LC activity, subsequent NA terminal release, amplitude, and dynamics, these peptides can elicit neuronal and circuit plasticity to enable dynamic changes in physiology and behavior. This peptide-mediated tuning likely functions in accordance with situational requirements such as in degrees of small, intermediate and hyper arousal. As such, it is likely that the gain control exerted by the LC onto its downstream pathways to optimize arousal levels for peak behavioral performance is mediated by these peptidergic inputs.

Several remaining fundamental conceptualizations of LC function need to be addressed before we fully grasp the functional significance of peptide-mediated modulation of the LC and its target regions. For example, peptidergic release is posited to be associated with phenomena that greatly increase intracellular calcium, in some cases being shown with stimulation at higher frequencies, activation of G_q_-coupled GPCRs, and photostimulation with more calcium-permeable opsins.^243^^,^^281^^,^^316^, ^317^, ^318^, ^319^, ^320^, ^321^, ^322^ The behavioral and physiological events that drive peptidergic input to the LC remain largely unknown. Progress in in vivo peptide detection,^280^^,^^282^ coupled with monitoring of NA activity using dual-color imaging systems^323^ or single unit recordings,^32^^,^^324^ could further elucidate how these signals are integrated and encoded. Likewise, we are still largely unaware of the excitatory/inhibitory balance between convergent and divergent inputs when it comes to LC regulation, as one might expect with convergent incoming NPY or opioids versus CRF signaling.^325^ The presence of multiple GPCRs for the same peptide within the LC/ peri-LC further complicates this matter. This complex expression pattern could lead to either excitation or inhibition of the circuit, or some type of feedback, as noted for NPY.^159^ Whether there is a functional threshold for preferential receptor activation such as affinity for its peptide, differences in abundance, sensitization, or recycling dynamics, has yet to be clarified in a physiologically intact LC preparation.

Additionally, as recent technological developments will soon enable, we need to better determine whether NA/peptides actions are synergistic, additive, or antagonistic when coreleased at LC terminal sites. The relevance of neuronal activity-independent downstream GPCR signaling will be harder to discern with such relationship. Isolating individual peptidergic contributions, by leveraging conditional or inducible KO strategies (as done previously with galanin^117^), could significantly accelerate these types of ongoing efforts. Moreover, it will be imperative to map out efferent innervation from peptidergic LC neurons to delineate whether they target distinct terminal fields and/or release sites, allowing for discrete information broadcasting and ultralocalized neuromodulation.

Finally, we consider that peptide-associated heterogeneity of the LC can be leveraged to address clinical pathology, in particular neuropsychiatric and neurodegenerative disorders. It is well established that LC dysregulation triggers an array of disease states that span the entire arousal-cognition continuum.^326^^,^^327^ As current pharmacotherapy targeting the LC-NA system remains common but very rudimentary, elucidating the peptidergic influences exerted toward and by the LC could provide novel therapeutic entries against a multitude of interrelated pathologies, including anxiety,^12^ posttraumatic stress disorder,^328^ attention deficit hyperactivity disorder,^329^ addiction,^176^ and neurodegeneration-associated cognitive decline.^330^^,^^331^ By honing into the therapeutic potential of selective agents, agonists, antagonists, and allosteric modulators that target neuropeptidergic signaling^332^ we may be able to transform our approach for modulating LC function and its output toward counteracting the effects of suboptimal noradrenergic transmission that contribute to pathology.

## Conflict of interest

The authors declare no competing interests.
